# D-MSR: A Distributed Network Management Scheme for Real-Time Monitoring and Process Control Applications in Wireless Industrial Automation

**DOI:** 10.3390/s130708239

**Published:** 2013-06-27

**Authors:** Pouria Zand, Arta Dilo, Paul Havinga

**Affiliations:** Pervasive Systems Group, Faculty of Electrical Engineering, Mathematics and Computer Science, University of Twente, P.O. Box 217, Enschede 7500AE, The Netherlands; E-Mails: a.dilo@utwente.nl (A.D.); p.j.m.havinga@utwente.nl (P.H.)

**Keywords:** distributed scheduling, real-time WSANs, WirelessHART, ISA100.11a, IEEE 802.15.4e

## Abstract

Current wireless technologies for industrial applications, such as WirelessHART and ISA100.11a, use a centralized management approach where a central network manager handles the requirements of the static network. However, such a centralized approach has several drawbacks. For example, it cannot cope with dynamicity/disturbance in large-scale networks in a real-time manner and it incurs a high communication overhead and latency for exchanging management traffic. In this paper, we therefore propose a distributed network management scheme, D-MSR. It enables the network devices to join the network, schedule their communications, establish end-to-end connections by reserving the communication resources for addressing real-time requirements, and cope with network dynamicity (e.g., node/edge failures) in a distributed manner. According to our knowledge, this is the first distributed management scheme based on IEEE 802.15.4e standard, which guides the nodes in different phases from joining until publishing their sensor data in the network. We demonstrate via simulation that D-MSR can address real-time and reliable communication as well as the high throughput requirements of industrial automation wireless networks, while also achieving higher efficiency in network management than WirelessHART, in terms of delay and overhead.

## 1. Introduction

Industrial control applications can be categorized into two main classes: (i) factory automation, and (ii) process control. Factory automation applications involve machines (e.g., robots) that perform discrete actions and are highly sensitive to message delays. Thus, such applications may require latency in the region of 2–50 ms. Process control, however, is typically used for monitoring and controlling the continuous production stream of fluid materials (e.g., oil and gas refinery) [1,2]. Due to the non-critical nature of the process control applications, latency requirements are not usually stringent (>100 ms) [1].

Based on the criticality and importance of the applications, the International Society of Automation (ISA) considers six classes of wireless communication, from critical control to monitoring applications, in which the importance of the message response time and Quality of Service (QoS) requirements vary [3]. In the more critical applications, sensor/process data need to be transmitted to the destination in a reliable, timely and accurate manner. Process control applications cover class 1 to 5 [1]. The details of the classes are shown in Table 1.

ISA100.11a [3] and WirelessHART [4] standards are designed for process control and monitoring applications. ISA100.11a supports industrial applications from class 1 to 5, and WirelessHART supports industrial applications ranging from class 2 to 5 [1]. Traditional wireless sensor networks (WSNs) are deployed in class 4–5 applications, in which low-power consumption is given priority over providing a bounded response time delay. ZigBee Pro [5], as one of the first standard for WSNs, is designed for applications which have softer real-time and reliability requirements, so it can not address the requirements of industrial control applications [6]. This paper will address the (i) real-time and (ii) reliable communication requirements of periodic monitoring and process control applications from class 1 to 5 in industrial harsh and dynamic environments. Those applications generally involve mostly static field devices (*i.e.*, sensors and actuators). Most of the traffic over the network consists of real-time sensor data that is published periodically (with a rate of 1 per second to 1 per hour [2]) toward the other sensors, actuators (in this paper, we assume that the actuators support a set of function blocks for controlling purposes) or the gateway for closed-loop process control and monitoring applications. In closed-loop process control applications (either critical or non-critical), the sensor data will travel in the field toward the actuator without involving the central control station, also called *control in the field*.

Certain Quality of Service (QoS) mechanisms are used by communication networks to meet the real-time requirements. These mechanisms can generally be categorized into: (i) *traffic classification* and (ii) *resource reservation*. The *traffic classification* mechanism can be used for channel access and packet delivery along the path between the endpoints, by labeling the packets with a priority value and placing them on the corresponding queue in the path. The *resource reservation* technique allocates the communication resources along the path between two end-points for a specific traffic or class of traffic to achieve the desired QoS requirement [7].

In addition to real-time communication, reliability is also an essential requirement for communication in harsh industrial environments in the presence of interference. The links quality between a source and destination node can heavily influence the success of the delivery of sensor data to the destination when the application needs it. Several mechanisms exist to increase link reliability. A survey is given in [7]. One of the mechanisms used to improve link quality, by trying to eliminate or minimize interference, is channel hopping. It is a diversity technique that can help prevent external interference and multipath fading [8]. Channel hopping technique is used in several industrial 802.15.4-based [9] standards such as WirelessHART, ISA100.11a and IEEE 802.15.4e (Time Slotted Channel Hopping (TSCH) mode [10]). IEEE 802.15.4e is a MAC amendment of the existing standard 802.15.4-2006 designed for low power and low bandwidth reliable communication in industrial environments.

Existing industrial wireless technologies such as WirelessHART and ISA100.11a use a centralized network management approach. While a centralized approach can generate optimal results for static networks, it has several drawbacks. Firstly, the network manager is prone to a single point of failure. In case of failure or network partitioning, nodes that do not have access to the network manager are left without management functionality. Secondly, the centralized approach incurs a high communication overhead and latency for exchanging management traffic. Lastly, they cannot cope with network dynamicity in a timely manner. These problems are exacerbated as the network scales up. We show in this paper that these problems are significant and we demonstrate how they can be solved.

This paper presents a Distributed Management Scheme for Real-time applications (D-MSR) that is built for wireless industrial automation. Using a distributed approach, D-MSR could address the issues of high throughput and reliable communication as well as real-time requirements, while achieving higher efficiency in network management in terms of delay and overhead. Issues such as node joining, reserving communication resources for exchanging management messages, constructing end-to-end connections between sensors and gateway/actuators for addressing real-time requirements, handling of network dynamicity such as node or edge (in this paper “edge” means a node-to-node connection in the network layer) failures, and data delivery in case of lossy networks, are all addressed by D-MSR.

According to our knowledge, this is the first distributed management scheme based on the IEEE 802.15.4e standard (TSCH), which supports the whole protocol stack and manages the nodes in different phases, from joining to publishing the sensor/process data in the network. Related work mainly focused on the data link layer that provides data delivery service in a timely and reliable manner in multi-hop wireless networks, which are discussed in Section 3.2.2.

To address all the listed issues, we define different mechanisms and modifications in different OSI layers. In the data link layer, we define a two-hop neighborhood schedule-matrix that is used to construct a communication schedule between different pairs of network devices (the terms “network device” and “node” refer to a field device, such as a sensor and actuator, as well as router that improve network connectivity) in a distributed manner. In addition, two modules are defined in the upper data link layer: *neighbor connection manager* and D-SAR. The *neighbor connection manager* defines initial communication links between each node and its neighbors. Therefore, the upper layers can use these primary links to communicate with a particular neighbor. In order to reserve communication resources and provide real-time communication between two end-points based on the required bandwidth, we use the D-SAR protocol [11]: a Distributed Scheduling Algorithm for Real-time applications based on concepts derived from Asynchronous Transfer Mode (ATM) networks [12]. The distributed nature of our resource reservation scheme, makes it feasible to change the reservation based on possible changes in the network connectivity, caused by the interference and dynamic link quality between the devices, in a timely manner. This capability together with the Clear Channel Assessment (CCA), re-transmission and channel hopping schemes in the data link layer, provide reliability in the network. As a response, D-MSR can address both the real-time and reliable communication requirements in a harsh industrial environment.

In the network layer, we use RPL (Routing Protocol for Low power and Lossy Networks [13]). In the transport layer we define, the *end-to-end connection manager* that establishes connections to either enable management communications (e.g., network layer control messages between network devices and gateway) or sensor/process data communications, through the D-SAR protocol. The sensors publish periodic data to actuators (the term “sensor to actuator”, “peer-to-peer” and “point-to-point” communication are used interchangeably) for process control applications, or to gateway for monitoring applications. In case of node or edge failures, the *end-to-end connection manager* releases the previously allocated communication resources along the old path, reserves new resources (in this paper “resources” means communication resources in the network) and establishes a new connection between the pairs through the new path, by applying the D-SAR protocol.

We compare via simulation the performance of D-MSR with that of WirelessHART (given the similarities between WirelessHART and ISA100.11a, the same result can be obtained by comparing D-MSR with ISA100.11a) in a typical industrial environment with high packet losses. We evaluate the end-to-end data delivery delay and compare the communication schedule and network throughput of D-MSR with that of WirelessHART. Furthermore, we evaluate the relationship between the packet delivery ratio and increased internal and external interference in the network. We show that in case of extensive external interference, D-MSR requires less time to reach a stable data delivery ratio value in comparison with WirelessHART. We compare the power consumption in the D-MSR network with that of WirelessHART. We show that by applying D-MSR, we can achieve higher efficiency in network management in terms of latency and overhead during node joining, resource reservation, end-to-end connection establishment, and when coping with dynamic situations (e.g., node or edge failure).

Section 2 provides background information about the MAC layer that is used in D-MSR. Section 3 describes D-MSR protocol stack architecture. Section 4 provides details about the functional description of D-MSR algorithms in different protocol layers. We provide details on the different phases of a network node from joining to publishing its sensor data, in Section 5. Section 6 elaborates on performance evaluation for real-time communication schedule construction, network throughput, data delivery in case of lossy networks, and management efficiency (in terms of delay, communication overhead), by comparing D-MSR with WirelessHART performance. Finally, Section 7 concludes the paper and summarizes our future research in this area.

## 2. Time Slotted Channel Hopping (TSCH) Concepts

Time Synchronized Mesh Protocol (TSMP) [14], developed by Dust Networks, is a media access and networking protocol that is designed for low power and low bandwidth reliable communication. TSMP concepts are used in several existing industrial wireless technologies such as WirelessHART, ISA100.11a and IEEE 802.15.4e (TSCH mode). IEEE 802.15.4e (TSCH mode) is a MAC amendment of the existing standard 802.15.4-2006. It enables robust communication through channel hopping. TSCH is based on a time-slotted mechanism, where a schedule dictates on what slot and which channel a node should transmit/receive data to/from a particular neighbor. TSCH does not address routing issues, but leaves this to the upper layers.

**Figure 1.**
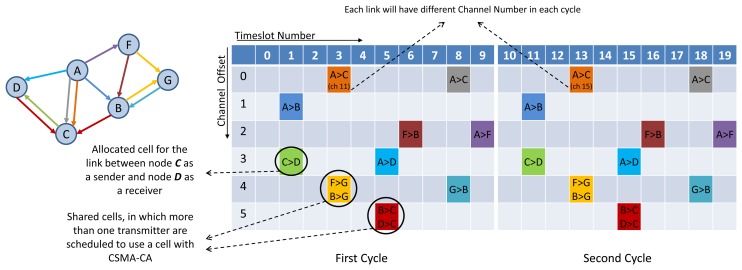
TSCH Slot-channel matrix (right) for the network shown on the left.

TSCH divides the wireless channel into time and frequency. Time is divided into superframes, which have a collection of discrete time slots. Figure 1 shows the TSCH matrix (e.g., for the network shown on the left) and illustrates a superframe of 10 slots. The size of a timeslot in TSCH is typically 10 ms. A link is a transaction that occurs within a cell. Link information consists of a superframe ID, source and destination IDs, a slot number referenced to the beginning of the superframe, and a channel offset. The simplest version of a link contains one transmitter and one receiver. The two nodes at either end of the link communicate periodically once in every superframe. If only one transmitter is scheduled, the cell is contention-free (in this paper, we focus on the procedure of constructing the communication schedule in contention-free (not shared) or dedicated/exclusive cell). A slotted CSMA approach can be used if multiple transmitters are scheduled to compete in a shared cell for transmitting to the same device simultaneously. Multiple links can be allocated from one node to another in different cells. For example, in Figure 1 two Tx-links from node A to C are shown. TSCH links hop pseudo-randomly over a set of predefined channels, one packet at a time. Each time a transmission is going to occur on a link, both sides of the link calculate the radio channel of the communication by taking “*(Absolute Slot Number + Channel offset) % Number of channels*” and by mapping the result to the related superframe frequency hopping pattern. *Absolute Slot Number* (ASN) is the number of timeslots since the beginning of the network, *Channel offset* is the link's channel offset in the matrix of slot-channel, % indicates modular division, and *Number of channels* is the number of available channels.

**Figure 2.**
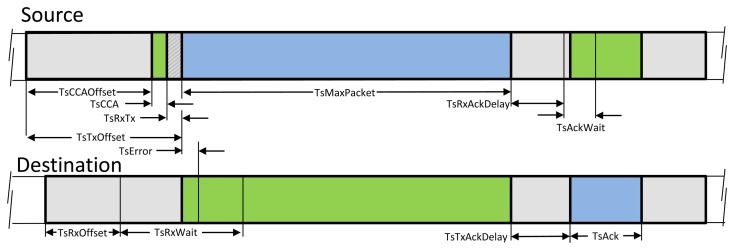
Timing of a dedicated TSCH timeslot.

Figure 2 depicts the specific timing requirement inside a TSCH timeslot. The scheduled communication in a timeslot between two nodes relies on an accurate time synchronization across the network. The network devices should have the same notion of when each timeslot begins and ends. TSCH, unlike the IEEE 802.15.4 that uses the beacon-based synchronization scheme, relies on exchanging timing offset information of the received and sent packets to provide synchronization. Each device should periodically synchronize its network time with at least one of its neighbors that is selected as a time synchronization source. The new device initially becomes synchronized with the network during the joining process and remains synchronized in the node's normal operation during any communication in a timeslot with a time source neighbor. Whenever a node receives an ACK with time correction information or a data packet from its times sources neighbor, it can adjust its clock. The mechanisms for time synchronization are described in [10].

**Figure 3.**
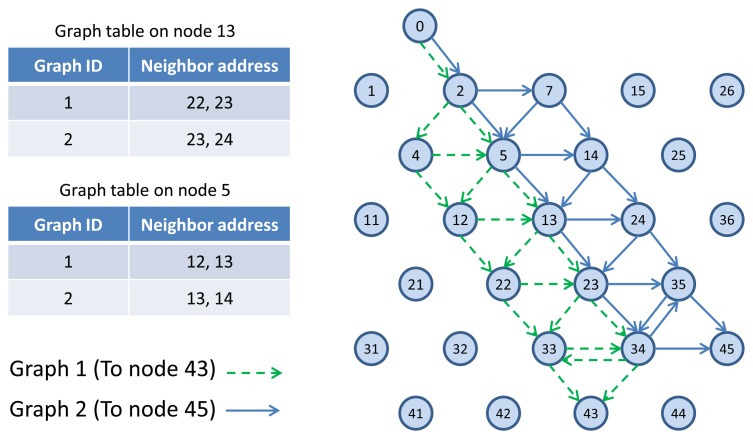
Graph routing sample.

TSMP works based on graph routing schemes. A graph is a routing structure that establishes directed end-to-end connection among devices. Each destination has its own graph, and several sources can share the same graph. Each graph in a network is identified with a unique Graph ID. Figure 3 illustrates the graph routing. In this figure, node 0 uses Graph 1 and 2 to communicate with nodes 43 and 45 respectively. When a source node wants to send a packet to a destination, a Graph ID will be included in the packet header to enable routing to the destination. At any node in the path, multiple next hops could be specified in a mesh graph; path diversity is directly built-in [14]. In Figure 3, for example, an intermediate node 5 may forward a packet identified by Graph 1 to node 12 or 13 and may forward a packet identified by Graph 2 to node 13 or 14. WirelessHART and ISA100.11a standards are designed by using concepts derived from TSMP. In those standards, graph-based routing is used, where the network manager constructs all the graphs/routes in the network, and applies them to the network devices.

**Figure 4.**
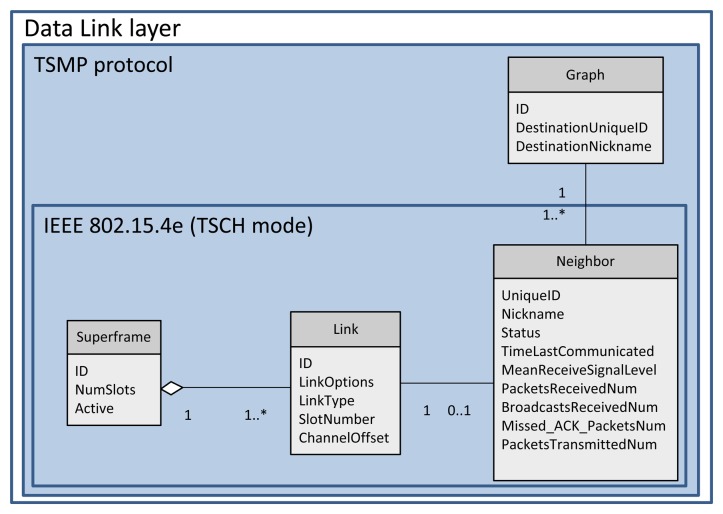
Communication tables stored in each device and their relationship in TSCH and TSMP.

A collection of communication tables is defined to enable communication and to control communication performance. These tables are configured by the network manager through the system manager module in each device via MAC layer service primitives. These tables and their relationships are shown in Figure 4 and described below:

- *Superframe table:* This table contains a collection of superframes. Based on the required communication schedule, multiple superframes of different length can be configured for each device by filling in this table. The practical superframe length is defined as 2*^n^* sec(−2 ≤ *n* ≤ 9) from 250 ms (2^−2^ sec) to 8 min and 32 sec (2^9^ sec) [15].
- *Link table:* This table contains a collection of links. This table, together with the superframe table, identifies the communication schedule. Based on the traffic rates, multiple links are scheduled for each device in different periods (by specifying the superframe ID to which the link belongs). Each link is specified by the node address, timeslot, channel offset, link type (Normal, Join, Discovery or Broadcast) and link option (Tx-link, Rx-link, or Shared Tx-link).
- *Graph table:* In a graph table, each graph represents the list of potential next-hop neighbors that the data can be forwarded to. This table, in collaboration with the route table located in the upper layer, provides sufficient information for routing the packets. TSCH does not address routing issues, but leaves this to the upper layer. Therefore, the graph table is excluded from D-MSR stack protocol.
- *Neighbor table:* Unlike the other communication tables, this table is not filled by the network manager. The neighbor table contains the list of neighbors the device can communicate with.

## 3. D-MSR Protocol Stack Architecture

**Figure 5.**
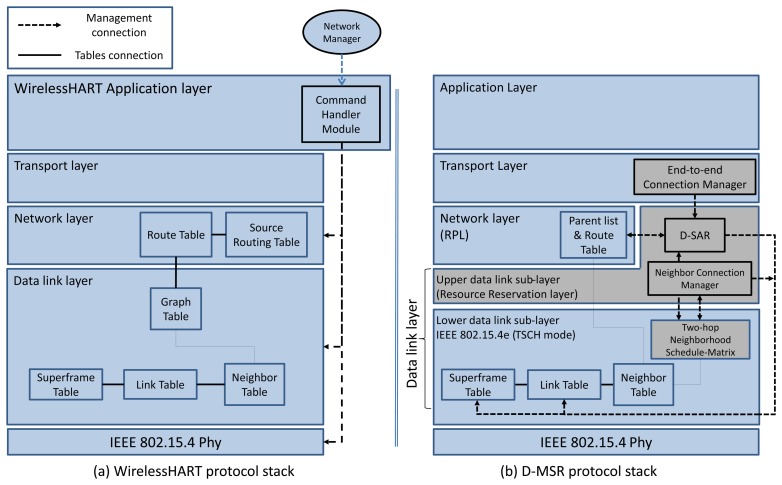
WirelessHART (**a**) and D-MSR (**b**) protocol stack.

In WirelessHART and ISA100.11a, a central network manager schedules all the network communications, constructs all the routes, and establishes end-to-end connections in the network. The protocol stack of WirelessHART, the connection between tables in different layers, and the managing procedures are shown in Figure 5(a). The network manager configures the communication tables in the data link layer and the routing table in the network layer through the *system manager module* implemented in each device. WirelessHART uses graph routing as well as source routing [4] in the network layer and use the Route Table and Source Route Table.

In D-MSR the network setup is performed in a distributed manner. This requires the implementation of various mechanisms in different layers. The D-MSR protocol stack is shown in Figure 5(b), in which the new sub-layers, modules and tables are displayed in a different color. The data link layer consists of two sub-layers: the lower and the upper data link sub-layer. In the lower data link sub-layer, we use the IEEE 802.15.4e (TSCH mode) standard, after having modified it to fit our requirements. A two-hop neighborhood schedule-matrix is added in this layer in order to schedule interference-free communications in the network. The modification details are discussed in Section 3.1. The upper data link sub-layer (the resource reservation layer) supports several features and functionalities which are normally data link layer functions, but are not currently included in the lower data link sub-layer. In this sub-layer, we implement D-SAR and *neighbor connection manager* modules that configure locally the communication tables in the lower data link sub-layer. These two modules use the information provided by the schedule-matrix to construct interference-free schedules in different network operation phases. These two modules are discussed in more detail in Section 3.2. The *end-to-end connection manager* module is implemented in the transport layer. This module establishes the end-to-end connection through the D-SAR protocol. The modifications carried out in the lower data link sub-layer, the upper data link sub-layer, routing layer, and transport layer, as well as the ways in which they can work together, are discussed in Sections 3.

### 3.1. Lower Data Link Sub-Layer

In the centralized approach the network manager constructs the communication schedule in line with the network devices requirements based on the global knowledge it has obtained from the network. For instance, the network manager in WirelessHART maintains a global schedule-matrix to keep track of the timeslot-channel cell usage by the network devices. Allocation of an interference-free cell to one pair of neighbor devices is feasible since the network manager manages the usage of that cell by any other pairs (the term “interference” refers to the “internal interference” caused by the concurrent transmissions in the same channel in the network). In addition, the network manager avoids spatial reuse of that cell in the network. However, in the distributed approach we need a distributed management scheme to avoid allocating the same cell to another interfering pair of devices, either in the network or neighborhood. The interference models can generally be classified into: (1) *physical* and (2) *protocol* interference model [16,17]. In the *physical* model, the feasibility of an interference-free communication is determined by the signal-to-interference ratio (SIR) of a receiver. In the *protocol* model, the feasibility of an interference-free communication is determined based on graph neighborhood relationship. In this paper, conform the *protocol* model, a node uses information about the allocated cells in its two-hop neighborhood to reserve interference-free cells, after which it will monitor the status of its scheduled cells to guarantee interference-free communications. To this end, a two-hop neighborhood schedule-matrix (the terms “schedule-matrix” and “two-hop neighborhood schedule-matrix” are used interchangeably) is defined in the lower data link sub-layer, in which each node maintains the current usage of its two-hop neighborhood cells. Each entry in the schedule-matrix represents the cell usage at that timeslot on that channel and is specified by the node addresses of the scheduled link. In order to establish initial links and to enable further communication between neighbors in different network operation phases, the neighboring nodes need to find the same unused cell in their schedule-matrices. The procedure for constructing and updating the schedule-matrix are discussed later on in Section 4.1.

**Figure 6.**
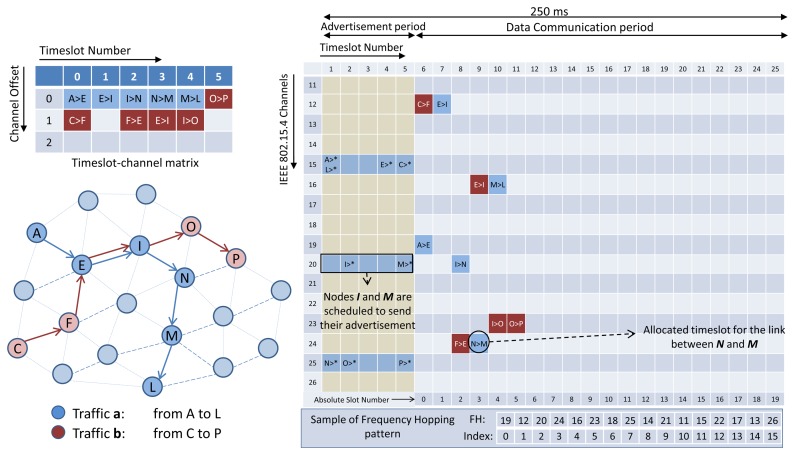
Modified Superframe.

In D-MSR, we use an idle listening to update the schedule-matrix in each node. The nodes listen to their one-hop neighbors advertisements to update their schedule-matrices. To this end, the advertisement (in TSCH nodes broadcast advertisements to enable network formation and to exchange timing information) also includes additional information about the subset of the advertiser link table (*i.e.*, node address, timeslot, channel offset, and superframe ID) that are used by the receiver to construct and update its schedule-matrix. Furthermore, in TSCH it is assumed that the network manager schedules the advertisement links between the advertiser and its neighbors. In order to assure that in D-MSR the node can hear their neighbors advertisements, we modified the TSCH matrix in the lower data link sub-layer by defining two periods in the superframe; the *advertisement period* and the *data communication period*. In the *advertisement period*, nodes either send their advertisements or listen to their neighbor advertisement. No further communication links are scheduled allowing for more data sharing between the nodes in the advertisement period. The additional information that is included in the advertisement is listed in Table 2. In the *data communication period*, communication schedules are reserved to enable the communication between neighboring nodes. Figure 6 shows this setup. The figure illustrates a superframe with a length of 250 ms consisting of 25 slots.

In the *advertisement period*, the nodes can choose a free advertisement cell in channels 15, 20, and 25 (these three channels do not overlap with any of the three common IEEE 802.11 channels. Therefore, less interference occurs in these channels. A similar concept is used in the ISA100.11a standard, in which these three channels are designated as slow hopping channels for purposes such as neighbor discovery) to send the advertisements. We limit the number of channels in that period to three, in order to facilitate neighbor discovery and data sharing during joining. If a node chooses a timeslot to send the advertisement, the node will transmit an advertisement in the assigned channel most of the time. If not, it listens in a randomly selected channel (after having chosen from three advertisement channels) to receive other neighbors advertisements. However, if a node is not supposed to send the advertisement in a timeslot in the advertisement period, the node will once again listen in a randomly selected channel. The procedure of selecting a free advertisement cell will be discussed in Section 4.1.

In the *data communication period*, D-MSR schedules interference-free communication links between the neighboring nodes. For example, as is shown in Figure 6, traffic ‘a’ and ‘b’ are transmitted from node A and C toward node L and P respectively. The scheduled communication for these traffic flows are shown in the slot-channel matrix in the top left of Figure 6. Each time a scheduled communication is going to occur on a link, both sides of the link calculate the radio channel of the communication by taking *“(Absolute Slot Number + Channel offset) % Number of channels”*. For instance, nodes I and N that select the timeslot 2 and channel offset 0, follow the frequency hopping pattern with that offset in the *data communication period* and will use channel 20 in that timeslot, as is shown in the *data communication period* (blue cells for traffic ‘a’ and red cells for traffic ‘b’) of the superframe. The procedure of scheduling the communication links (*i.e.*, filling the link table and superframe table) in the *data communication period* are handled by the *D-SAR* and *neighbor connection manager* modules in the upper data link sub-layer, which are discussed in Sections 3.2.

### 3.2. Upper Data Link Sub-Layer (Resource Reservation Layer)

To enable the initial communication between two neighbor nodes (that can be used by the routing layer), these nodes should agree on the same link (timeslot and channel offset). Furthermore, based on the traffic that passes through this edge, more links need to be reserved to enable real-time end-to-end connections. In the centralized approach, the network manager schedules the initial communication resources as well as the required resources for further communications and fills in the data link layer communication tables in each network device based on those schedules. However, TSCH does not describe any distributed mechanism, by which either the initial communication links for neighbor nodes or more communication resources, for real-time end-to-end communications, can be allocated. For this reason, we define an upper data link sub-layer (resource reservation layer) on top of the data link layer, to configure the data link layer communication tables and to schedule the communications between neighbors.

Two modules are defined in the upper data link sub-layer: *neighbor connection manager* and the D-SAR module. The *neighbor connection manager* allows the TSCH MAC protocol to be glued onto the higher layer (routing layer), besides providing initial neighbor nodes communications. The D-SAR module reserves communication resources along the path in different phases of the network operation to enable real-time end-to-end connection either for management traffic purposes or to sensor/process data traffic. As is shown in Figure 5(b), *neighbor connection manager* and D-SAR modules configure the data link layer communication tables (the link table and superframe table), to allocate or release the communication resources. The remainder of this section focuses on the *neighbor connection manager* and D-SAR module respectively.

#### 3.2.1. Neighbor Connection Manager Module

TSCH does not describe how the communication links should be constructed to enable initial communication of a node with a particular neighbor. However, the next upper layer (network layer) that resides on top of TSCH, assumes that nodes are capable of communicating with all their neighbors. In response, the *neighbor connection manager* (in the upper data link sub-layer) defines the initial communication links (one Tx-link and one Rx-link) between each device and its neighbors. This can be done by adding new links and superframes in the link table and superframe tables. The relation between the *neighbor connection manager* and communication tables in the lower data link sub-layer is shown in Figure 5(b).

In order to establish the initial communication links between neighboring nodes, they need to agree to communicate in a particular interference-free cell. To this end, a handshaking mechanism is needed between the new device and each of its neighbors to choose the common unused cell (*i.e.*, timeslot number and channel offset). The details of handshaking mechanism are discussed in Section 4.2.

#### 3.2.2. D-SAR Module

Real-time control applications require data to be transmitted over long distances through a multi-hop network in a reliable and timely manner. However, most recent studies [18–21] on data link layer use the centralized resource reservation (scheduling) scheme to provide timely and reliable data delivery service. The centralized scheduling schemes have several disadvantages. They often perform poorly in terms of reaction time, as all updates need to be sent first to the base station for further processing. A distributed resource reservation algorithm is needed which would allow source nodes, based on the requirements of the application and traffic characteristic, to reserve network resources for its peer communications along their paths for addressing different QoS needs. Relevant techniques from other networking-related domains (e.g., Asynchronous Transfer Mode (ATM)) could potentially be adapted to develop solutions that are suitable for wireless sensor and actuator networks [7].

D-SAR is a distributed scheduling algorithm that is based on concepts derived from ATM networks. This is because the ATM signaling protocols [11] also address performance issues in terms of reliability and timeliness of packet delivery.

The D-SAR protocol is used to establish an end-to-end connection (for supporting point-to-multipoint or point-to-point traffic) and to reserve the communication resources based on the traffic characteristics requested by the source node, along the path toward the destination in different phases of the network operation. These traffic flows can be either network management traffic (e.g., network layer control messages) or sensor data traffic that are published periodically by the sensor nodes toward actuators or gateway. The D-SAR module in the upper data link sub-layer receives the request for establishing a connection from the *end-to-end connection manager* in the transport layer. The D-SAR module in each device, reserves and releases the communication resources by modifying the link table and the superframe table in the lower data link sub-layer. The relation between the D-SAR module and the *end-to-end connection manager* and communication table is shown in Figure 5(b).

Before initiating the D-SAR protocol, the network is already established, all nodes have joined thenetwork, the initial communication links have been established between neighbor nodes, and the routing layer has constructed the routes between network nodes. The details of the D-SAR protocol are discussed in Section 4.3.

At different phases of the network operation, the D-SAR protocol allocates or releases the communication resources (links and superframes), based on a request that may be initiated either from the upper layers in the stack or received from the other neighbors. In Sections 5.4 (Phase-4), 5.5 (Phase-5), and 5.6 (Phase-6) the details of these procedures are explained.

### 3.3. Routing Layer and Transport Layer

We use RPL in the routing layer. RPL is designed for Low power and Lossy Networks (LLNs), which consist of nodes with limited capabilities, such as processing power, memory, and battery power. RPL is defined for a network, in which nodes interconnections are lossy and the traffic rate is low [13]. These characteristics make RPL suitable for use in wireless industrial networks.

**Figure 7.**
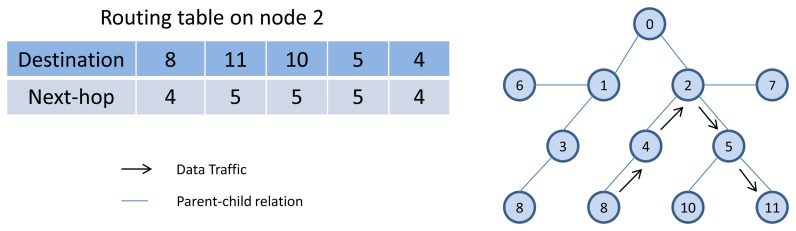
Point-to-point traffic model in RPL.

RPL is a distributed routing protocol that supports the up, down, and point-to-point traffic model by forwarding the packet to its selected parent from the parent list, based on the objective function (for example, by selecting the parent with the best Expected Transmissions values in the up direction) or by selecting a neighbor form the routing table as a next hop (in the down direction). The parent list and route table in the network layer, and their relationship to the neighbor table in the data link layer are shown in Figure 5(b). In the point-to-point traffic model, when a node (e.g., a sensor) needs to reach another node (e.g., a actuator), its packet travels in the “up” direction toward a common ancestor and is then forwarded down toward the final destination. For example, as is shown in Figure 7, node 8 needs to communicate with node 11. The packet first travels “up” toward node 0. However, in the “up” route toward the root, the packet reaches node 2, which is a common ancestor between node 8 and 11. Node 2, which contains the destination address of the packet in its routing table, then forwards the packet toward node 11 through node 5.

In the transport layer, the *end-to-end connection manager* establishes the management connection (between new devices and the gateway) as well as an end-to-end connection (between sensors and gateway/actuators) through the D-SAR protocol. In the case of node or edge failure, the connection manager releases the previously allocated resources along the old path, and re-establishes a new connection by allocating new resources along the new path.

## 4. Functional Description of D-MSR Algorithms in Different Protocol Layers

In this section, we first illustrate the mechanisms used to select the advertisement cell and to construct schedule-matrices in the lower data link sub-layer. Next, we discuss the mechanism that is used to define the initial communication links with neighbors in the upper data link sub-layer. Finally, we explain the D-SAR protocol used to establish an end-to-end connection and to reserve the communication resources in the upper data link sub-layer.

### 4.1. Selecting Advertisement Cell and Constructing Two-Hop Neighborhood Schedule-Matrix

To let a new node choose the free advertisement cell in a distributed manner, the new device should listen to its neighbor's advertisement. The advertisement includes the advertisement cell numbers of a node and its neighbors. This effectively allows a receiving node to gather advertisement cell information about its two-hop neighborhood. The new device then chooses a free advertisement cell based on this information. A similar scheme is proposed to allocate the timeslot in a distributed manner in [22].

**Figure 8.**
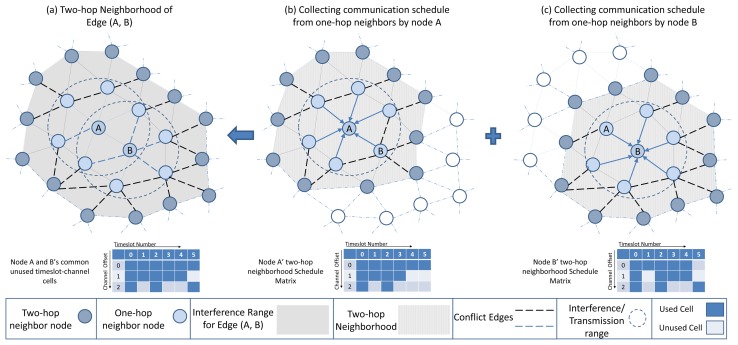
Selecting an interference-free cell on (**a**) edge (A, B) based on the constructed schedule-matrix of (**b**) node A and (**c**) node B.

In the *protocol* interference model, the transmission on one edge (e.g., between node A and B) is interference-free and can only be activated in one timeslot-channel cell if there is no transmission on any edge that disturbs either A or B, as is shown in Figure 8(a). The conflicting edges (shown by black dashed lines) with edge (A, B) can be formulated, based on the [16] model, as follows:

(1) $$\text{ConflictSet}((A,B))=\{(C,D)\in E|[\{C,D\}\cap \{A,B\}=\emptyset \text{]}\wedge [\{A,B\}\cap (R_{C}\cup R_{D})\neq \emptyset ]\}$$

*ConflictSet* denotes the set of conflicting edges with the edge (A, B). The set of all edges in the network is denoted by *E* while *R_C_* denotes the set of nodes that are possible receivers of node *C*. In addition to the *ConflictSet*, other edges (shown by blue dashed lines) that are sharing a node with edge (A, B) cannot be scheduled in the same cell. That is because we assume that each node has a single radio transceiver and cannot simultaneously receive and transmit. In a realistic setting, the interference and transmission range of a node may not be equal. However, in D-MSR we assume, for simplicity, that the interference and transmission range of a node are equal. In case these ranges are not the same, considering an additional *virtual* edge representing the interfering edges [16] can be a possible solution. The details of *virtual* edge mechanism are discussed in Section 5.6.3.

Each node maintains a schedule-matrix to keep track of the current cell usage in its two-hop neighborhood, as shown in Figure 8(b,c) for nodes A and B. The schedule-matrix is constructed based on the link table information that the node collects from its one hop neighbor's advertisements. In the received link table information from one hop neighbor, the links between the one-hop and two-hop neighbors are included. Any two nodes that wish to establish an interference-free link with each other can negotiate based on their schedule-matrix and find a common cell that is not used by any of their possible conflict edges in their own two-hop neighborhood.

### 4.2. Defining Initial Communication Links with Neighbors

The idea of defining the initial communication links with neighbors is derived from [23]. In [23] the authors describe the algorithm that provides the initial communication link between a mobile node and its adjacent neighbors. Mobile nodes change their connectivity very rapidly as a result of which the reservation of communication resources and the provision of real-time communication between two end-points are not considered. However, by modifying this algorithm based on our requirements, the initial communication links between a node and all of its adjacent neighbors can be scheduled. In [23], while the nodes are trying to schedule the communication links with their neighbors, they choose a random channel offset for each link and use that channel for their further communications on that link. However, assigning a different channel offset to conflicting edges is not discussed in [23]; to handle internal interference, nodes need to ensure that while communicating nodes choose the same frequency, conflicting edges use different channels. Moreover, in [23] the advertisements are sent on channel 0 and all the neighbor nodes listen on channel 0 in their free timeslots to receive the advertisements. As the nodes schedule fills up, they spend less time listening on channel 0 for advertisements. This means that nodes with more busy schedules have difficulties adding more bandwidth. D-MSR allows for more data sharing between nodes by considering the special period in each superframe for sending advertisements.

We define five states: “*Aloha*”, “*Transmit Connection Request*”, “*Receive Connection Request*”, “*Transmit Data*”, and “*Receive Data*” for each timeslot, as in [23]. The default state for all the timeslots in the *data communication period* is *Aloha.*

**Figure 9.**
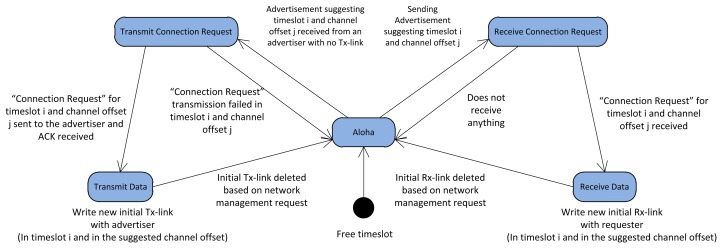
Different state of sample timeslot in the *data communication period*.

Figure 9 illustrates different states of a sample timeslot in the *data communication period*. At the beginning of each superframe, each node sends an advertisement in the scheduled advertisement cell in the advertisement period. This advertisement includes free timeslots, *i.e.*, the timeslots with *Aloha* state in the *data communication period.* To assure interference-free communication, the advertisement suggests for each free timeslot an unused channel offset chosen from the free cells in the timeslot column at the schedule-matrix. After sending the advertisement, the advertiser changes the state of these free timeslots from *Aloha* to *Receive Connection Request* state, and listens for a potential *Connection Request* from the neighbors in the suggested channel. A neighbor node that receives the advertisement, checks whether it has any timeslot with *Transmit Data* state with the advertiser or not. If not, the neighbor tries to find a common unused timeslot-channel cell with the advertiser. Once found, it converts the selected timeslot state from *Aloha* to *Transmit Connection Request*. The neighbor sends a *Connection Request* to the advertiser in the selected timeslot-channel entry. By receiving the *Connection Request* packet, the advertiser changes the state of that timeslot from *Receive Connection Request* into *Receive Data* and sends the acknowledgement of receipt to the neighbor. Upon receiving the acknowledgment, the neighbor changes the state of the selected timeslot from *Transmit Connection Request* to *Transmit Data*. If no *Connection Request* is received by the advertiser, the state of that timeslot is changed to *Aloha*. This procedure continues until the new node has established one timeslot with *Transmit Data* state and one timeslot with *Receive Data* state with all of its neighbors. Subsequently, a new node writes interference-free links in the communication tables, one Tx-link and one Rx-link for each of its neighbors. The channel offsets and timeslots of these links are set to the negotiated timeslot-channel entries, and the typical superframe (the length of the initial superframe is assumed to be 2 seconds) is added to the communication tables.

### 4.3. D-SAR Protocol

The *end-to-end connection manager* in the transport layer of a source node, which intends to establish a connection, sends the connection-request to the D-SAR module in the stack, including the connection parameters such as a destination address, traffic/connection ID, connection priority (we use the same priority of data as defined in the WirelessHART protocol for exchanging the management, sensor data, alarm, or normal packets), communication type (periodic or non-periodic), and a requested publishing period. In this paper, we assume the prevalence of periodic data traffic between sensors and actuators.

**Figure 10.**
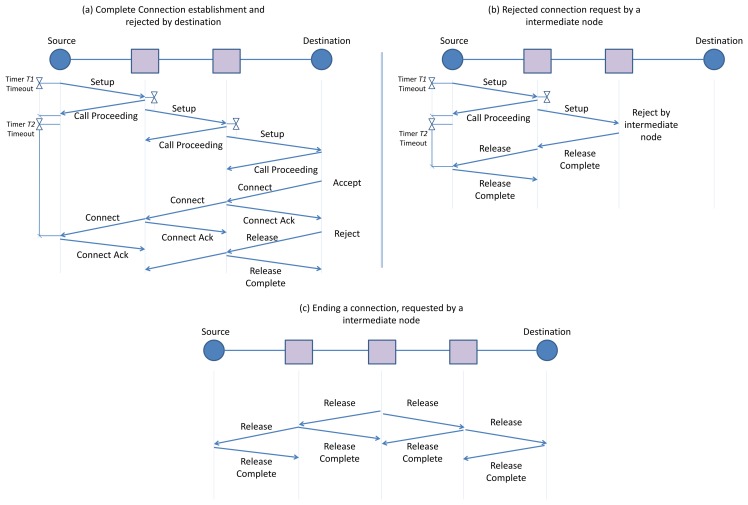
Overview of connection establishment protocol.

The D-SAR module at the source node initiates the procedure by sending a *Setup* message to the next hop toward the destination along the route defined by the routing layer. The *Setup* message includes parameters such as a list of suggested common unused timeslot-channel cells for further communication with the next hop, a destination address, traffic ID, timeslot-channel cell selected on previous hop (the information about the timeslot-channel cell selected by the previous hop, is used by the next hop in order to minimize the end-to-end delay), and a requested publishing period. The sender selects these common unused cells based on the received information about the next hop link table (by listening to the next hop advertisement) and its own schedule-matrix. The receiver of the *Setup* message then performs a check of its available communication resources. The receiver checks whether any of the suggested cells are unused in its own schedule-matrix with the requested publishing period. It also checks if there are unused cells with the requested publishing period to communicate with the next hop. If the required resources are available, the receiver chooses one cell from the suggested free cells and allocates the requested communication resource based on the requested publishing period of the traffic by writing a new link and (if needed, new) superframe in the related tables in the data link layer. The receiver will then respond by sending the *Call Proceeding* message that includes the chosen cell. In the next step, the receiver (intermediate node) forwards the *Setup* message toward the destination node with some delay. This delay enables the neighbors to update their schedule-matrices based on this new reservation that will be published in advertisements, thereby avoiding conflicts over resource reservation. This process continues until the destination node receives the *Setup* message as shown in Figure 10(a). However, at any intermediate node the receiver of the *Setup* message can refuse the connection request with a *Release Complete* message if it is unable to accommodate the new connection as shown in Figure 10(b).

The destination node can either accept or decline the new connection request from the source node by sending the *Connect* message or *Release Complete* message. This *Connect* message traverses along the multihop network back to the source node. All the temporary communication resources, which are reserved during the *Setup* message exchanging, are switched to permanent reservation. This two-step reservation is performed to ensure that timeslot reservations are not carried out should the connection request be unsuccessful.

After establishing the connection and during the network operation, either the source node (e.g., because the connection has expired or is no longer required), the intermediate node (e.g., because of node/edge failure, changing the route or detecting the conflict in the reserved resources), or the destination node may wish to end the connection. The node that wishes to end the connection transmits the *Release* message toward the end(s) of the connection. The procedure of ending a connection by an intermediate node is shown in Figure 10©. The receiver of the *Release* message deletes the communication schedule established with the sender and a *Release Complete* message is sent to the sender. The communication schedule is specified by the traffic ID. Next, the receiver of the *Release* message forwards the *Release* message to the next hop in the route toward the end-point of the connection. Upon receiving the *Release Complete* message from the next hop, it will then delete the communication schedule constructed with the next hop, which is specified by that traffic ID. This process continues until the *Release* message reaches the end-point of the connection. This procedure ensures that all nodes along the route release all the resources previously allocated to the connection. In this case, the D-SAR module in each node deletes the related links and superframes from the communication table. The details of the D-SAR protocol for the source, the intermediate, and destination node are provided in [24].

## 5. D-MSR Management Phases

**Figure 11.**
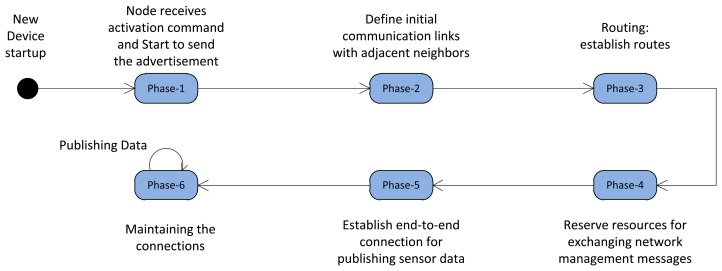
Different states of a node operation in the network.

In this section we discuss the different management phases, which guide the new node from startup to the moment the node starts to publish/subscribe the periodic sensor data in the network. The node operation state machine is shown in Figure 11.

After a new node startup, in Phase-1 the node receives the activation command from the neighboring advertiser and starts to send the advertisement. In Phase-2 the initial communication resources between the node and its adjacent neighbor are allocated, by which the routing layer in Phase-3 can establish the routing graph. In Phase-4, the required communication resources should be allocated in the network to exchange the management messages in the routing layer. After construction of the routing graph and allocation of management resources, the end-to-end connection can be established between the sensors and actuators/gateway to publish the sensor data toward the destination(s) which is done in Phase-5. At the network setup stage each node goes through these phases. This procedure continues until all the devices have joined the network and started the operation. During normal operation of the network, in Phase-6, the D-MSR maintains the end-to-end connections by coping with dynamicity, by handling the resources reservation conflict, and by coping with internal and external interference. The following sections discuss these phases in more detail.

### 5.1. Receiving an Activation Command and Starting to Send the Advertisement (Phase-1)

**Figure 12.**
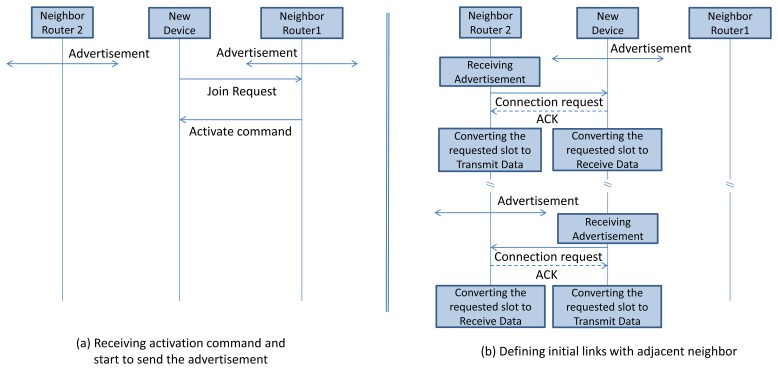
Receiving activation command (**a**) and defining initial communication links with neighbors (**b**).

The new device that intends to join the network listens on a physical channel for a period of time and then continues on the next channel, until all the channels have been scanned. The new device selects the best advertiser/candidate according to predefined criteria and sends the join request to the selected advertiser. In this work we select the advertiser according to the Link Quality Indicator (LQI) or Received Signal Strength Indicator (RSSI) of the received advertisement, although other criteria can be easily added. The advertiser sends the join response/activation command to the new device, upon acceptance (e.g., if the advertiser can still admit new devices). Sending the join request and receiving the join response procedure is implemented using the IEEE 802.15.4e standard. The joining procedure of a new device is shown in Figure 12(a).

Upon receiving the activation command, the new device starts to send the advertisement. However, before starting to send the advertisement, the new device should choose a free advertisement cell by listening to its neighbors advertisement (discussed in Section 4.1). The new device can choose a free advertisement cell based on this information and then start to send the advertisement in the advertisement period.

### 5.2. Defining Initial Communication Links with Neighbors (Phase-2)

After the new device joins the network, it needs to find the route toward the other nodes in the network or the gateway. The *neighbor connection manager* module in each network device, uses a handshaking mechanism (explained in Section 4.2) in order to define one Tx and one Rx link with each of its neighbors. Those links and a typical superframe will be added in the data link layer communication tables. These links enable a node to communicate with all its neighbors. Afterwards, the routing layer can be run to find the path between the endpoints. This procedure is shown in Figure 12(b).

### 5.3. Constructing the Routes (Phase-3)

In this phase, the routing layer finds the routes between the endpoints. In D-MSR we have used RPL in the routing layer. RPL specifies how the new device finds a path toward the gateway. By generating the RPL control messages, the routing entries in the intermediate nodes will be constructed as well as a complete path toward the new device. Several control messages, e.g., DAO (Destination Advertisement Object control message is used to construct routes to the other intermediate or leaf nodes) message, are forwarded through the network periodically to maintain and update the “up” (multipoint-to-point) and “down” (point-to-multipoint) routes.

### 5.4. Reserving Management Resources (Phase-4)

In this phase, the node reserves resources for exchanging network management messages. Once the node joins the network, in Phase-2 the initial communication links to adjacent neighbors are constructed and then in Phase-3 the routing layer constructs the “up” and “down” routes. In this phase, it is necessary to reserve the communication resources by which the routing layer control messages can be forwarded to the destination along the path. To reserve the management resources through the “up” path, each node runs the D-SAR protocol to allocate the required resources based on the DAO messages rate (which is defined in the routing layer). Similarly, to reserve the resources through the “down” path, the root runs the D-SAR protocol toward the new nodes.

In a centralized approach, such as WirelessHART or ISA100.11a, a different procedure is defined to receive the join request from the new device, send the activation command, construct the new graphs for the new device, and reserve the management resources (e.g., management superframes and links). In these standards, the join request will be forwarded toward the network manager via the proxy device, and the network manager who has received the join request will use its centralized algorithm to allocate the management communication resources (such as graphs, superframes, and links). In the centralized approach, a join response/activation command is sent to the device after all necessary communication resources for exchanging the management messages have been configured and reserved along the path. The joining sequence of a new device in WirelessHART network is discussed in [25].

In D-MSR we consider the node as having joined the network, after it received the activation command from the neighbor advertiser, started to send the advertisement periodically (Phase-1), defined the initial communication links with adjacent neighbors (Phase-2), constructed routes to the other nodes (Phase-3), and reserved the communication resources to exchange the management messages (Phase-4).

### 5.5. Establishing an End-to-End Connection for Periodic Sensor Data Communication (Phase-5)

Having allocated the initial resources, as well as the management resources, the focus of this phase is to establish an end-to-end connection between a sensor and an actuator or a sensor and the gateway for transporting the application data. C*ontrol in the field* (*i.e.*, closed-loop control through a peer-to-peer communication between a sensor as a publisher and an actuator as a subscriber. This is part of traditional Fieldbus technologies) is important for process control applications (see Class 1 in Table 1). WirelessHART networks support peer-to-peer communication between sensors and actuators only if the traffic is routed via the gateway. This is required from WirelessHART's security mechanism to prevent potential safety threats resulting from undetected and unmonitored communications [4]. ISA100.11a addresses control in the field by providing a secured peer-to-peer communication. D-MSR addresses real-time communication between sensors and actuators (providing control in the field) as well as between sensors and the gateway.

As we focus on applications that require constant data traffic rates, D-MSR allocates a virtual circuit for each traffic flow. This implies that the resources reserved for each end-to-end connection depend on the traffic characteristics requested by the source node. The source node initiates this phase by sending a *Setup* message as was shown in Figure 10. The format of this message is similar to the Request Service in WirelessHART and the Contract Request in ISA100.11a.

In WirelessHART, if the sensor node needs to have a connection with another device, which can be an actuator or gateway, it will send the Request Service to the network manager with specified bandwidth and latency characteristics. The network manager needs time to schedule new communications along an uplink graph from the sensor to the gateway, and from the gateway to the actuator along a downlink graph. It will then reply to the requesting node. The process of asking for more communication resources is discussed in more detail in [25]. However, unlike in ISA100.11a and WirelessHART, which both send the request to a centralized network manager, in D-SAR the source node sends the *Setup* message toward the destination node along the route defined by the routing layer in a distributed way.

**Figure 13.**
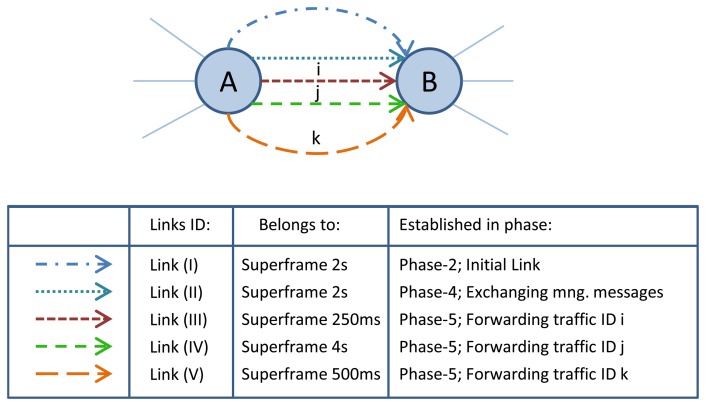
ATM concepts in link definition between nodes A and B.

The traffic ID parameter, which is included in the *Setup* message, is used to specify the allocated resources for that traffic ID. For example, in case of releasing the specific connection resources, the traffic ID is used to identify the related communication resources that are allocated for that connection. However, to allow for the efficient utilization of each link during the normal network operation, they are shared, upon their allocation, between multiple traffic flows rather than assigned specifically to one particular traffic flow. This means that the communication resources, which are reserved for initial communications, management communications, or different end-to-end connections between sensors and actuators, are shared between different traffic flows. For example, let us consider nodes A and B in Figure 13. Five links are established between the two nodes in different phases (e.g., link (II) that belongs to the superframe with 2 s length is established in Phase-4 for exchanging management messages, and link (III) that belongs to the superframe with 250 ms length is established in Phase-5 for forwarding traffic ID i). Using the ATM networking concepts, management traffic, traffic ID i, traffic ID j, and traffic ID k are allowed to use all the defined links between node A and B during the normal operation of the network.

### 5.6. Coping with Dynamicity, Reservation Conflict and Interference in the Network (Phase-6)

#### 5.6.1. Coping with Dynamicity in the Network

**Figure 14.**
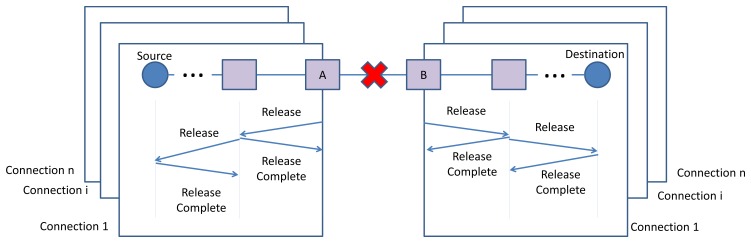
Releasing the allocated communication resources after edge failure.

In order to cope with network dynamicity, such as node or edge failure, the connection manager in a node (*i.e.*, incident nodes of the broken edge or adjacent nodes of the failed node that are part of end-to-end connections), transmits *Release* message(s) toward the source node(s) or destination node(s) by applying the D-SAR protocol. In case of edge failure, the incident nodes of the broken edge (node A and B in Figure 14) transmit the *Release* messages, including the traffic ID information, toward the end-points of each connection that passed through the broken edge.

The process of releasing the reserved communication resources, which is identified by the traffic ID, is executed for each of the connections containing the broken edge. At this stage, all the resources previously allocated to that connection will be released and become free. This means that the related links and superframes are deleted from the communication tables of each device in the former route.

**Figure 15.**
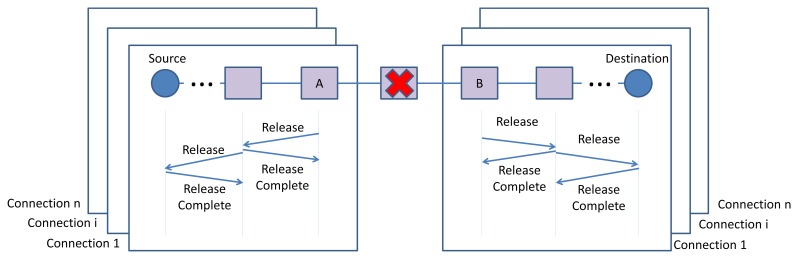
Releasing the allocated communication resources after node failing.

As Figure 15 illustrates, in case of node failure, the adjacent nodes which joining edges are part of an end-to-end connection, release the allocated resources by transmitting the release messages toward the sources or destinations of the end-to-end connections. Exchanging the *Release* and *Release Complete* messages and releasing the resources follows the same procedure of edge failure.

The routing layer repairs the former routes. Upon receiving the *Release* message, the connection manager in the source node will re-establish a new connection and reserve the new resources along the new path once more by using the D-SAR protocol.

In case of a node or an edge failure in centralized approaches like WirelessHART or ISA100.11a, the failure should be reported to the network manager. Subsequently, the network manager establishes new routes, releases the previous communication schedule, and constructs new schedules.

#### 5.6.2. Handling the Resource Reservation Conflict

**Figure 16.**
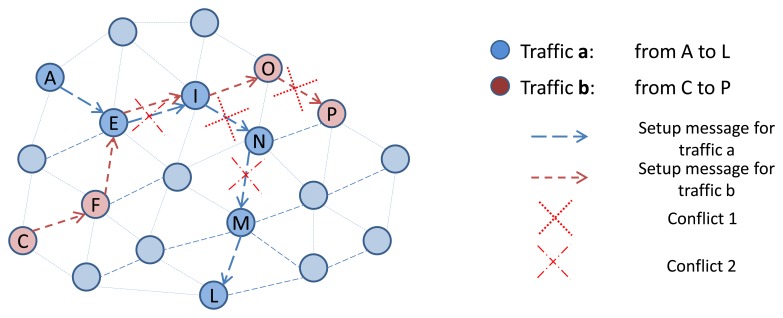
Resource reservation conflict sample.

In D-SAR protocol, the two nodes of an edge participating in end-to-end connections, negotiate to reserve a common unused timeslot-channel cell based on their current two-hop neighborhood schedule-matrix. By considering the intentional delay before forwarding the *Setup* message, we allow their neighbors to update their schedule-matrix based on the new reservation. However, there is still a probability that a conflicting edge may also choose that cell, prior to receiving the new advertisements listing the changes in their neighborhood schedules. The nodes of the conflicting edges that have reserved the same cell should handle this conflict upon detection (the detection is done by observing the constant packet loss in that cell), by releasing the conflicting reserved resources. As a response, the end-to-end connection manager in the node transmits *Release* messages toward the end-points of the connection that include the conflicting cells. Figure 16 illustrates these two potential reservation conflicts scenarios. In the first scenario, when the *Setup* message (e.g., for traffic a) is being forwarded along the path, the same cell is chosen by edge (E, I) and its interfering edge (N, M). That is because node N did not receive the node I advertisement to update its schedule-matrix based on the new reservation on edge (E, I). This possible conflict is avoided in D-SAR protocol by the considered intentional delay in forwarding the *setup* message. The second conflict happens, when two setup messages (that belonged to two different end-to-end resource reservation) choose the same cell simultaneously in conflicting edges (I, N) and (O, P).

#### 5.6.3. Coping with Internal Interference in the Network

**Figure 17.**
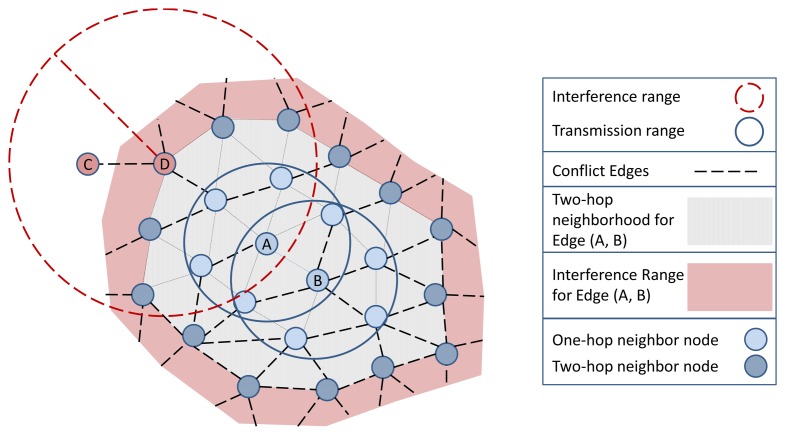
Internal interference caused by communication on edge (C, D) outside of the two-hop neighborhood of edge (A, B).

In a realistic setting, the interference range of a node may be much larger than its transmission range. Concurrent transmission in the same cell may cause interference even when the edges are two hops away from each other. Figure 17 illustrates how the communication on edge (C, D) that is outside of the two-hop neighborhood of edge (A, B) interferes with edge (A, B). Thanks to the scheduled communications concept, internal interference caused by communications outside of the two-hop neighborhood, happens in specific timeslot-channel cells that can be recognized by (1) observing the constant packet loss in those cells after reservation or (2) by performing CCA before reservation. By considering the *virtual* links that represent the interfering links, adding these in the schedule-matrix and by subsequently avoiding to use those timeslot-channel cells, the internal interference can be solved in a distributed manner. In Section 6.5.1, we evaluate the effect of this scheme in improving the packet delivery ratio in case of internal interference in the network.

#### 5.6.4. Coping with External Interference in the Network

In case of interference in the network, different edges may experience a different packet loss ratio. In centralized approaches like WirelessHART, each node periodically reports on the status of its communication with its neighbors to the network manager through a set of report commands. The network manager may re-construct new graphs, which include more reliable edges, based on the received reports from the network. It then releases former resources and constructs new communication schedule along these new graphs. These instructions will be forwarded to the network. This approach cannot cope with disturbance in large-scale networks in a real-time manner.

However, in D-MSR, the RPL uses best Expected Transmissions values (the expected number of transmissions required to successfully transmit and acknowledge a packet on the edge), as a metric, to find the best paths in case of interference. Subsequently, after choosing the new path, the previous resources along the old path are released, and the new communication resources will be reserved along the new path in a distributed manner. In Section 6.5.2, we compare the performance of D-MSR to that of WirelessHART in terms of the ability to provide reliable communication in case of interference in the network.

## 6. Performance Evaluation

This paper has discussed the distributed management scheme ability to serve applications requiring a real-time and reliable communication as well as a high throughput. This section illustrates how these requirements are fulfilled. To this end, we first assess the end-to-end data delivery delay of D-MSR and WirelessHART. Next, we evaluate the communication schedules and network throughput. Following this, we assess the packet delivery ratio in case of internal and external interference. Furthermore, the power consumption in the D-MSR network and WirelessHART is being evaluated.

Finally, we evaluate the management efficiency of D-MSR in terms of delay and overhead during node joining, management resource reservation, end-to-end connection establishment, and coping with changes and disturbances in the network.

### 6.1. Implementation of D-MSR and WirelessHART in NS-2

We implemented the D-MSR protocol stack in NS-2. In the data link layer we implemented IEEE 802.15.4e (TSCH mode). In the routing layer we implemented RPL in NS-2.

We also implemented the WirelessHART protocol in NS-2 [25]. It is the first implementation that supports the WirelessHART network management algorithm as well as the whole protocol stack of the WirelessHART standard.

### 6.2. Simulation Model, Parameters and Network Topology

**Figure 18.**
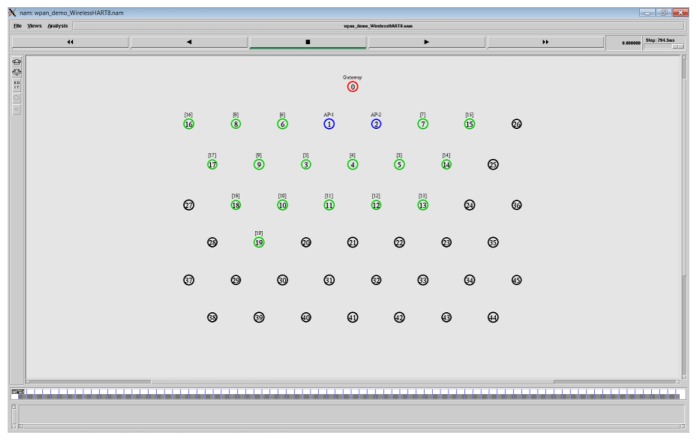
The network topology from animation tool of NS-2 simulator (nam).

In the simulations we set a network area of 100 m × 100 m, the transmission range of 15 meters, and neighbors distance of around 10 meters. We use the *two-ray ground model* as a radio propagation model. The network consists of 46 wireless nodes that are evenly distributed in the simulation area. The network topology is shown in Figure 18. This regular topology helps to evaluate the behavior of D-MSR and WirelessHART more accurately. For instance, in Section 6.5.2, we can evaluate the effect of increasing interference regions on the data delivery ratio rather precisely, by controlling the number of edges that were affected by interference in each step.

The length of management superframes, which is defined to allow for the exchange of management messages, is set to be 2 seconds. All the obtained results are based on the 2 seconds management superframes. In addition, in D-MSR, the size of link table entries, which are included in the advertisement payload, may reach 400 bytes. In the simulations, we assume that these amounts of data can be compressed in the advertisement payload with a size of 100 bytes. The detailed parameters are presented in Table 3.

### 6.3. Real-Time Evaluation

**Figure 19.**
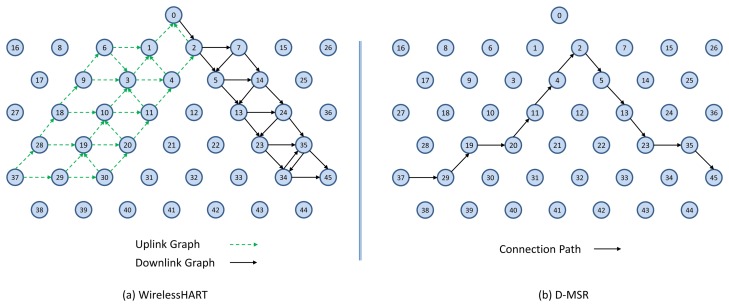
End-to-end connection between nodes 37 and 45 in WirelessHART (**a**) and D-MSR (**b**).

To evaluate the end-to-end data delivery delay, 29 pairs of sensors and actuators were considered in the network. These pairs are chosen in such a way that the total hop distances of the sensor to the gateway and of the gateway to the actuator are spread in different hop levels. In Figure 19 a sample of an end-to-end connection is shown between a sensor node (37) and an actuator node (45) based on WirelessHART and D-MSR network, respectively.

**Figure 20.**
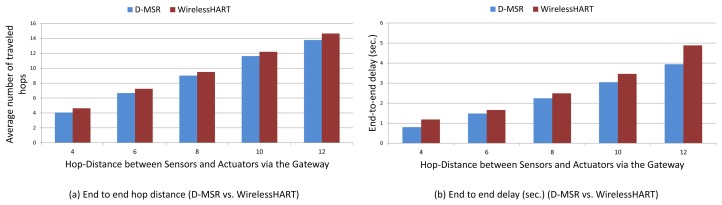
Average end-to-end hop distance (**a**) and delay (**b**) (D-MSR *vs.* WirelessHART).

We evaluate the average end-to-end data delivery delay and the average number of hops that the received packets need to travel to reach their destinations through the 29 connections. The results are shown in Figure 20 for both D-MSR and WirelessHART. In this figure, we classified connections into five categories based on the total hop distance of sensor to actuator via the gateway. We forward the traffic (periodic sensor data) from sensors towards actuators, by employing the constant bit rate (CBR) traffic model in NS-2 for all end-to-end connections. The requested publishing period of the sensor data for all 29 connections is set to two seconds. Subsequently, communication resources are reserved to exchange sensor data messages between the sensors and actuators/gateway based on that period.

The end-to-end delay in D-MSR is close to that of WirelessHART, which implies that D-MSR achieves similar results in addressing the real-time requirements during an operational phase. In those connections, in which the periodic sensor data packets have to travel more hops to reach their destination, more end-to-end delay is expected.

### 6.4. Network Throughput

**Figure 21.**
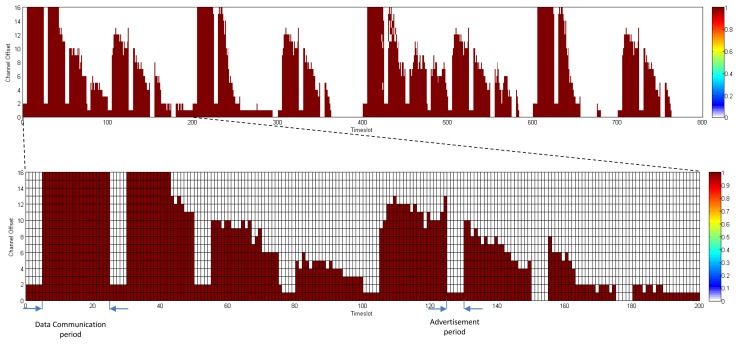
The global matrix of the current slot/channel usage (the advertisement period is also considered for the WirelessHART network to ensure a fair comparison of communication resources) for the sample WirelessHART network.

**Figure 22.**
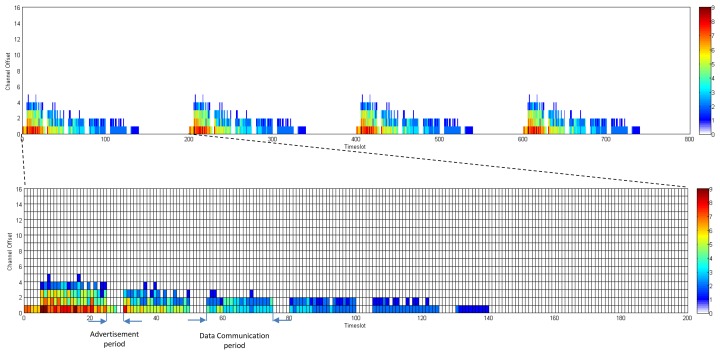
The combination of schedule-matrix of the current slot/channel usage for the sample D-MSR network.

In this section, we compare the communication schedule and network throughput of D-MSR with those of WirelessHART. Figures 21 and 22 show samples of constructed schedules for 29 end-to-end connections with a publishing period of two seconds in WirelessHART and D-MSR respectively. In these matrices, the communication schedule reserved for transmitting either management traffic or sensor data are shown. Through different colors in each cell in the matrix, the number of edges re-using that particular timeslot-channel cell are shown. Figure 21 shows the global matrix (the combination of superframes with a size of 25, 200, 400 and 800 timeslots) of the allocated timeslot-channel cells by the WirelessHART network manager. In this scenario, the network manager schedules each communication in an interference-free cell and avoids the spatial reuse of any cell between different edges, except for during the advertisement period. Figure 22 shows the combination of all schedule-matrices in each node in the network, which represents the global schedule-matrix (the combination of superframes with a size of 25 and 200 timeslots) in D-MSR. The D-MSR matrix looks more dense with more unused cells. There are two reasons for this. Firstly, in D-MSR the nodes just keep track of current cell usage in their own two-hop neighborhood. This means that reuse of the same cell in different two-hop neighborhoods could occur. As is shown in Figure 22, a given cell may be reused by 10 edges in different neighborhoods. Secondly, since more edges are considered in the uplink and downlink graph, more communication schedules are constructed in the WirelessHART network.

**Figure 23.**
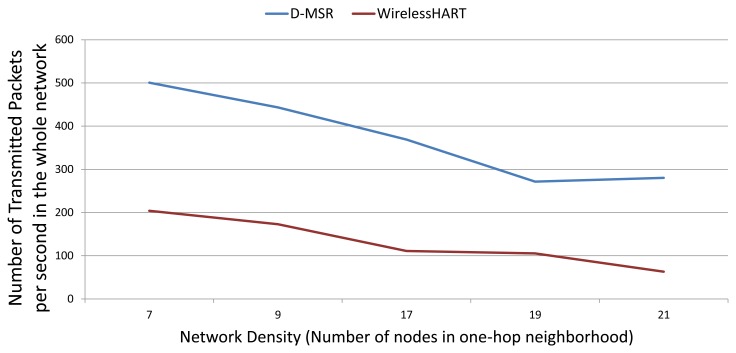
Network throughput (D-MSR *vs.* WirelessHART).

In addition, we evaluate the network throughput of both D-MSR and WirelessHART in different network densities. We gradually increase the transmission range of nodes in five steps from 15 to 25 meters to provide a different network density from seven to 21 neighbors in the one-hop neighborhood. For each network density, to evaluate the reachable network throughput, we try to establish the maximum number of end-to-end connections between field devices. As the network density increases, more bottlenecks are observed and less end-to-end connections can be established. In D-MSR more end-to-end connections can be established thanks to the RPL in the routing layer which does not need to route the traffic through the access points. On the other hand, the implemented WirelessHART passes all the traffic through the gateway. Figure 23 shows the network throughput: the number of transmitted packets in the whole network per second. As the network density is increased, the network throughput for both D-MSR and WirelessHART decreases, but less severely so for WirelessHART.

The spatial reuse of communication resources provides more network throughput for D-MSR than WirelessHART in the case of a sparse network. For example, when the number of neighbors in the one-hop neighborhood is seven, the network throughput is around 150% higher for D-MSR than WirelessHART. However, as the network becomes more dense, less disjoint two-hop neighborhoods can be observed. Consequently, less communication resources can be reused, which results in less network throughput difference. The communication schedule of D-MSR and WirelessHART for each of those five densities are shown in the Appendix. In the case of D-MSR, the communication schedules become more sparse and more channels are allocated to the communication schedules, as network density increases. In summary, the spatial reuse of communication resources in D-MSR improves the throughput in the large-scale network.

### 6.5. Reliability in the Network

Several techniques are used in industrial technologies to ensure reliable wireless communication, such as re-transmission, channel hopping, and multipath routing. The re-transmission scheme depends on the re-transmission of failed packets. In case of errors, this scheme incurs significant communication overhead as well as additional latency in delivering the packets. In multipath routing technique, each node has multiple next hops to forward the packet. When interference causes disruption of communication between a node and its next hop, an alternative path can be used to transport data [7]. Channel hopping and re-transmission schemes are used in the data link layer of D-MSR and WirelessHART. The multipath routing technique is deployed in the WirelessHART standard. In this section, we first evaluate the packet delivery ratio in case of internal interference as well as the effect of re-transmission capability. Next, we assess the behavior of D-MSR and the WirelessHART routing mechanism in terms of reliability in case of extensive external interference. To this end, we set up a number of experiments to evaluate the performance of data delivery in case of lossy networks.

#### 6.5.1. Data Delivery Ratio in Case of Internal Interference

**Figure 24.**
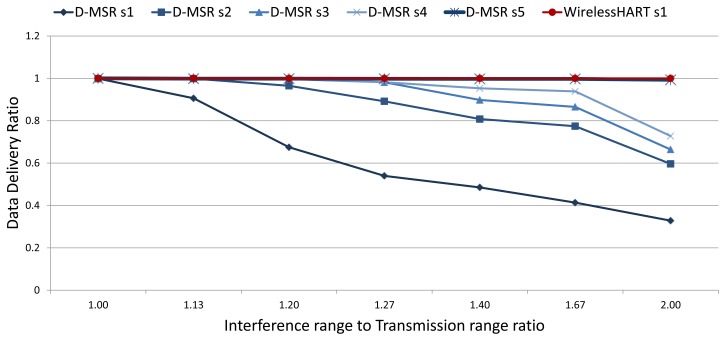
Data delivery ratio differences in case of internal interference.

In the previous evaluations, we assumed that the interference and transmission ranges are equal and that the two hops reuse distance guarantees interference-free communication in one cell. However, in a realistic setting, the interference and transmission range of a node may not be equal. To address this issue, we evaluate the relation between packet delivery ratio and increased internal interference in the network, in the first experiment. We define five scenarios in D-MSR. In order to assess the worst-case scenario, in the first scenario (D-MSR s1), D-MSR deliberately does not attempt to release the interfered communication link and the MAC re-transmission is not used. In the second, third, and forth scenarios (D-MSR s2, s3, and s4), the re-transmission with one, two, and three retries limit is used in the MAC layer. The fifth scenario (D-MSR s5) combines the advantages of both the re-transmission scheme and the *virtual* link method (discussed in Section 5.6.3). For WirelessHART, we have one scenario (WirelessHART s1) that does not use MAC re-transmission. Figure 24 shows the data delivery ratio for those six scenarios (D-MSR s1-5, and WirelessHART s1), in case of different interference to transmission range ratios. It is noticeable that the increase in interference range causes more internal interference in the network thereby decreasing the data delivery ration in D-MSR s1-4. On the other hand, D-MSR s5 and WirelessHART s1 provide more reliability in coping with internal interference in case of different interference ranges. This is because D-MSR s5 combines the retransmission by *virtual* link method, while WirelessHART s1 avoids the spatial reuse of communication resources.

In summary, the spatial reuse of communication resources in D-MSR (without considering the re-transmission techniques and *virtual* link method) is prone to reduced reliability due to internal interference.

#### 6.5.2. Data Delivery Ratio in Case of Lossy Network

**Figure 25.**
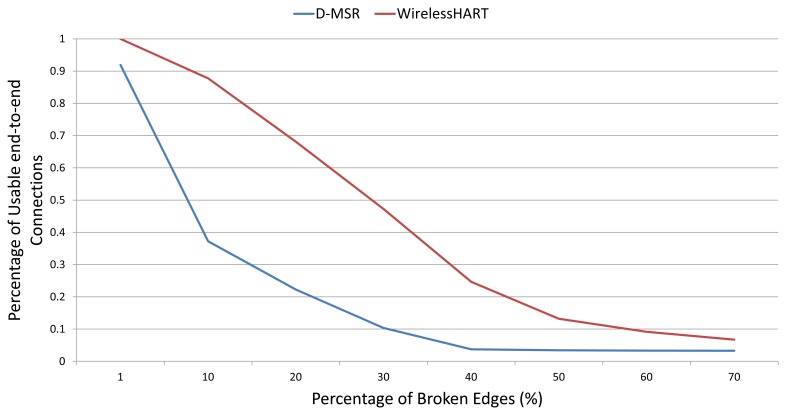
Reliability of the end-to-end connections.

In this section, we evaluate the behavior of the D-MSR and the WirelessHART routing mechanisms in terms of reliability. In the second experiment, we assume that the edges can only have two states, namely working or failed. We first increase the percentage of broken edges in the network and then measure the number of connections (from the 29 connections that were defined in the previous section) that are still working, *i.e.*, connect the sensors to the actuators. Figure 25 shows that in the WirelessHART network, thanks to its multipath routing scheme, more than 50% of the connections are still usable upon increasing the percentage of broken edges to 30%. In contrast, for D-MSR we have around the same, 50% loss of end-to-end connections, when only 10% of edges are broken.

However, thanks to the distributed nature of D-MSR, it can cope faster with interference (or edge failures) than WirelessHART, which uses the centralized approach. There is, therefore, a trade-off in applying the multipath routing in WirelessHART and in applying the distributed scheme to cope with the interference.

In the third experiment, we evaluate the relationship between packet delivery ratio and increased interference in the network. To do so, we forward the CBR traffic (periodic sensor data) from sensors towards actuators, for all the 29 connections. At the destinations/actuators we then measure the number of received packets. Unlike in the earlier experiment outlined above, we now assume that the quality of edges may vary from 0% to 100%. In this experiment, we gradually increase the interference regions in the network in six steps (each step takes 2,000 seconds).

**Figure 26.**
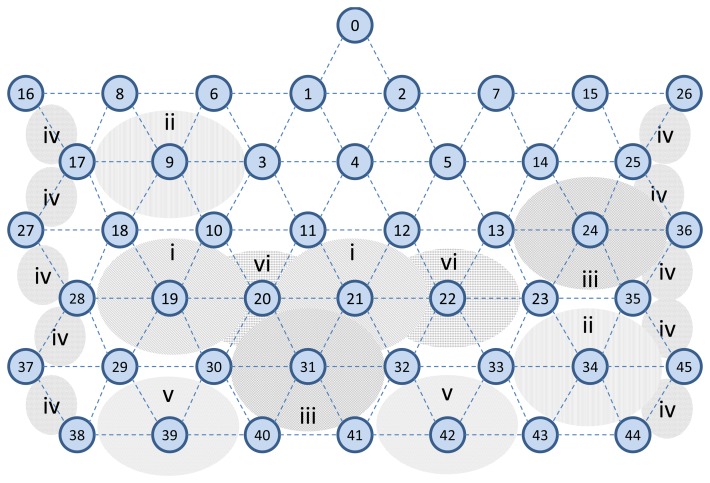
Applying interference incrementally in six steps.

These six steps of applying interference in the network are shown in Figure 26 and listed in Table 4. In each region, we randomly apply a different interference value to the edges between the nodes. This is because in a realistic harsh environment, each device may experience various packet loss ratios during the communication with each of its neighbors, which may be caused by external interference, non-line of sight connections, multipath fading or the shadowing effect. In this experiment, we assume that the more interference applied to an edge, the higher the chance that the packets will get lost.

We define two scenarios in D-MSR. In the first scenario (D-MSR s1), we gradually increase interference in the network in six steps, while the sensor data are forwarded between the sensor and actuators pairs. Following this, we measure the packet delivery ratio of all the connections between the sensor and actuator pairs. In this scenario, in order to assess the worst-case scenario, D-MSR deliberately does not attempt to re-construct the routes and re-schedule communication.

In contrast, in the second scenario (D-MSR s2), the routing layer adjusts the affected routes, the D-SAR protocol releases the previous allocated resources on the affected paths and reserves new resources along the new path. Meanwhile, the data are being forwarded through the connections and the packet delivery ratio of all the connections between the sensor and actuator pairs are being measured.

For WirelessHART we define three scenarios. The first scenario (WH s1) operates under similar conditions as that of D-MSR s1 to also assess the worst-case scenario in WirelessHART. To this end, the WirelessHART network manager does not adjust the affected routes and each node selects its next hop randomly based on the WirelessHART protocol.

In the second scenario (WH s2), we assume that the condition of the poor interfered edge will be reported to the network manager conform the WirelessHART protocol. The network manager re-establishes new graphs through the least affected edges, releases the previously reserved resources on the old path, and then reserves new resources along the new graph/route. These instructions are forwarded towards the network devices upon filling the communication tables of the devices. In this scenario, each node selects the next-hop neighbor on the given graph in a random manner, in line with the WirelessHART protocol.

In the third scenario (WH s3), similarly to the second scenario, we assume that the network manager re-establishes new graphs. However, to pursue better data delivery ratios, each node chooses the best next-hop neighbor based on local information on the packet loss ratio of each neighbor. This mechanism is used in each node during data delivery, while the network manager is collecting information on the edges health status, re-establishing new routes, re-constructing new communication schedule, forwarding the new instruction to the network, and after the maintaining process is finished during normal operation of network.

**Figure 27.**
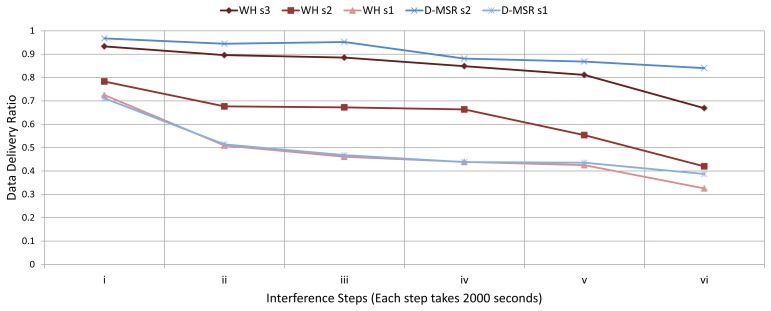
Data delivery ratio differences in five scenarios.

Those five scenarios (D-MSR s1, D-MSR s2, WH s1, WH s2, and WH s3) are shown in Figure 27 D-MSR s2, in which the routes are repaired and resources are re-allocated, performs better than WH s2, in which the network manager re-constructs the interfered graphs and nodes select the next hop in a random manner and even better than WH s3, in which the nodes select their best next-hop neighbors based on their local information (an action which is not listed in the WirelessHART protocol). For instance, after three interference steps have been applied in the network, the data delivery ratio measured is around 7.5% more for D-MSR s2 compared to WH s3 and 41% more compared to WH s2. This large difference between D-MSR s2 and WH s2 can be explained by two facts. Firstly, in WirelessHART more edges are defined in the uplink and downlink graphs to increase their reliability. If the interference in question is extensive in the network, the repaired graphs still may include some poor edges as well. Therefore, if the nodes randomly choose the next hop, these poor edges may also be selected by them. Secondly, the centralized nature of WirelessHART requires more delay and overhead to fix the problem in the network that greatly affects the data delivery ratio. However, WH s3, in which the nodes select their best next-hop neighbors based on their local information, outperforms the WH s2 regarding packet delivery ratio. Figure 27 does not consider the overhead of the repairing phase in terms of delays and the number of required communications.

As is shown in Figure 27, the performance of WH s1, in which the network manager does not repair the interfered edges and in which each node selects the next hop randomly, nearly matches that of D-MSR s1, in which the interfered edges and routes are not repaired. The fact that in WirelessHART more edges are defined in the uplink and downlink graphs, does not increase the probability of success in delivering the data in the case of extensive interference and random selection of next hop.

In summary, in the worst case scenario when the two protocols do not attempt to re-construct routes and re-schedule communication, D-MSR s1 performs close to the WirelessHART multipath routing mechanism (in WH s1). However, in the second scenario, the distributed approach (D-MSR s2) assures a higher data delivery ratio than WirelessHART (WH s2 and WH s3). As can be concluded from the first experiment, applying the multipath routing scheme in D-MSR, as a management scheme with a distributed nature, will provide more reliability in data delivery in case of link failures.

**Figure 28.**
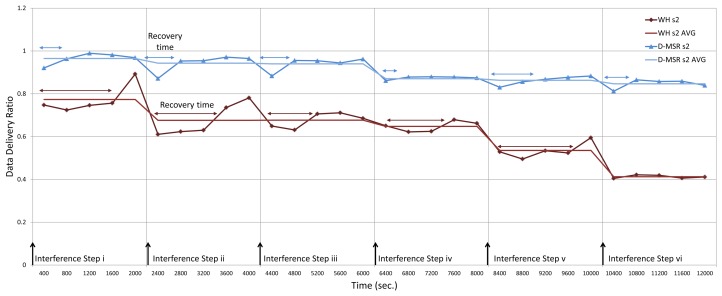
Data delivery ratio difference in two scenarios.

In Figure 28, we evaluate the repairing mechanism applied in D-MSR (D-MSR s2) and in WirelessHART (WH s2) to show which mechanism achieves a stable data delivery ratio the fastest. We consider five measuring points in each step to have more detailed view of data delivery ratio changes between interference steps. That way, we can show the behavior of each scheme in recovering the data delivery ratio after applying the interference in each step. As Figure 28 shows, the data delivery ratio suddenly drops each time the interference is applied in the network. As expected, D-MSR requires less time to reach the stable data delivery ratio value in comparison with WirelessHART. For instance, after applying the second interference step it takes more than 900 s for WirelessHART and around 500 s for D-MSR to reach a stable state value. This is because D-MSR needs less time to re-construct the new routes, to release the previous resources along the interfered route and to reserve new resources along the new path. In this figure, the AVG value in each step represents the average of the data delivery ratio in that step.

### 6.6. Power Consumption in the Network

In this section, we evaluate the energy-consumption of network nodes in D-MSR and WirelessHART. The simulation runs for 1,000 s. We measure the total consumed energy at every node during the simulation period. We consider two states of network operation, namely operation in (1) a static and (2) a dynamic environment (e.g., link failures). In the static environment we measure the energy needed to exchange network management messages (periodic updates), as well as application data messages (from sensors to actuators). For the dynamic environment we measure the energy consumed for the network maintenance.

The periodic management messages generated by each device in the WirelessHART network are network health reporting and status commands (*i.e.*, WirelessHART command 779, 780, and 787) and advertisements. In the D-MSR, each device broadcasts the advertisement packets, and the RPL periodically sends controlling messages to maintain routes. Management and application data messages for both D-MSR and WirelessHART are listed in Table 5.

Table 6 shows the energy-consumption (in this simulation we assumed the energy-consumption in Tx/Rx turnaround, and the processing energy can be neglected) required for each type of transaction. In addition, the idle listening energy at an unused scheduled link is shown. This is the energy that is consumed by the receiver while it is waiting for a message to arrive. Values of the parameters used in Table 6 formulas are listed in Table 7.

**Figure 29.**
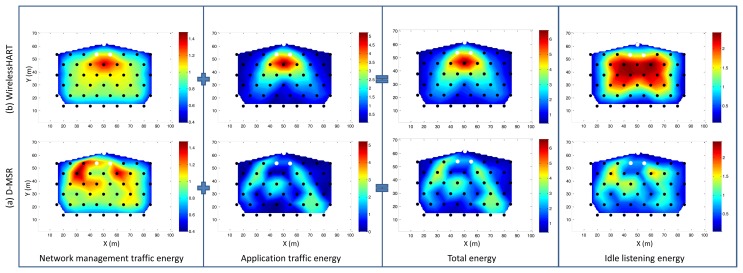
The energy consumption distribution in WirelessHART (**a**) and D-MSR (**b**).

The distribution map of energy consumption for network management traffic and application traffic in the case of static environment is illustrated in Figure 29. The total energy consumption over the network for management and application traffic is provided in Table 8. The total energy consumption for network management is higher in D-MSR than in WirelessHART, which can be explained by the higher data rate of control messages in RPL. From the network management energy map we can see that the distribution pattern for WirelessHART is symmetric, reflecting the regularity of the multi-path routing graph, with bottlenecks being the nodes close to the access points. The distribution pattern in the D-MSR management energy map, is also reflecting the structure of the RPL routing tree, with bottlenecks created at nodes close to the access points.

The application traffic energy map of WirelessHART shows bottlenecks close to the access points, which is due to the fact that all traffic should pass through the gateway. The energy consumption at bottleneck nodes in WirelessHART is higher than in D-MSR bottlenecks. The total energy consumption in WirelessHART is also higher than in D-MSR, which is due to the fact that RPL routing in D-MSR forwards traffic through shorter routes that do not necessarily pass via the gateway. Depending on the position of sources and destinations in the network, the bottlenecks in D-MSR can be more spread in the network area compared to the concentration of bottleneck nodes in WirelessHART. The distribution pattern in the total energy maps is more affected by the application traffic energy pattern.

Figure 29 also shows the energy consumption for idle listening. This energy depends on the efficiency of the scheduling mechanism. The better the scheduling, the less energy is needed for idle listening. As a response we did not include that energy in the total consumed energy in the network.

Table 8 also lists the consumed energy for network maintenance messages in case of 3–9 link failures. D-MSR requires less overhead and less maintenance energy for coping with disturbances (e.g., link failures) in the network. In Section 6.7.3, we evaluate the performance of D-MSR in coping with network dynamicity in more detail.

### 6.7. Evaluating Management Efficiency

#### 6.7.1. Performance During Node Joining

In this section we evaluate the procedure of node joining in D-MSR as well as in WirelessHART, in terms of delay and overhead. We group the nodes based on their distance from the gateway into six categories in our evaluation. In D-MSR, the joining delay for each node at different hop distances in phase 1–3 (*i.e.*, from the moment the node is started up till the node finds its path toward the gateway and the other devices) is the same. This is because most of the communications in phase 1–3 occur locally and do not depend on the hop distance of the node from the gateway. However, in Phase-4 as the hop distance increases, the delay in reserving the management resources increases as well. This is caused by the fact that in Phase-4, each node needs to reserve the management resources along the path towards the gateway and conversely. As the hop distance increases, the reservation procedure takes more time.

To compare our node joining procedure with that of WirelessHART, we consider the total delay and overhead during Phases 1–4 in D-MSR. This is because in WirelessHART, the nodes that have sent the join request to the network manager, must wait to receive the activation command from the network manager after all the necessary network management resources (such as graphs and communication links) have been configured and reserved along the path. The joining procedure in WirelessHART therefore consists of forwarding the join request towards the network manager, allocating the required management resources for all the nodes along the path, and finally forwarding the activation command towards the new device.

**Figure 30.**
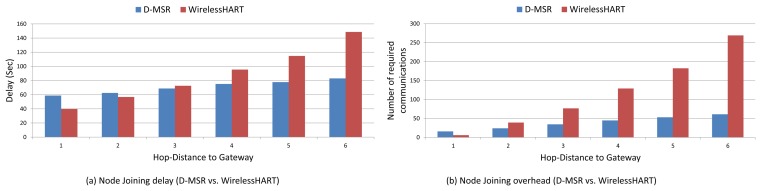
Nodes joining delay (**a**) and overhead (**b**) (D-MSR *vs.* WirelessHART).

Figure 30 displays, the delay in nodes' joining and the number of required communications (number of messages sent) for node joining in the case of different hop distance categories. It is noticeable that the increase in hop distance results in more delay, and in a larger number of communications for joining the nodes. They do so for both D-SAR and WirelessHART.

Figure 30 indicates that there exist considerable difference in the delay and number of required communications in node's joining between D-MSR and WirelessHART. It shows that the distributed scheme can perform far better in large-scale networks. It implies that D-MSR performs better in those scenarios in which the node joins and leaves the network frequently.

#### 6.7.2. End-to-End Connection Establishment between Field Devices

**Figure 31.**
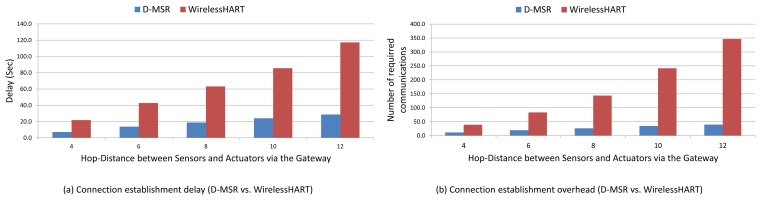
End-to-end connection establishment delays (**a**) and overhead (**b**) (D-MSR *vs.* WirelessHART).

In this section, we evaluate the management efficiency in reserving the communication resources and establishing end-to-end connections between 29 pairs of sensors and actuators. We classified connections into five categories based on the total hop distance of sensor to actuator via the gateway. Figure 31 displays, the delay in establishing connections (reserving communication resources) and the number of subsequent required communications for establishing those connections.

It is noticeable that the increase in the total hop distance of the pairs results in more delay, and in a larger number of communications for establishing connection. This is so for both, D-MSR and WirelessHART, but less severe for D-MSR.

Figure 31 indicates a considerable difference in the delay and the number of required communications between D-MSR and WirelessHART. For example, when the total hop distance of sensors to actuators comprises 12 hops, the average of the connection establishment delay is around 75% less for D-MSR compared to WirelessHART, while the average number of required communications for connection establishment is 88% less. Part of this difference can be explained by the fact that in WirelessHART, the network manager has to define more edges to provide a reliable uplink and downlink graph. Subsequently, more communication schedules have to be constructed for those graphs. As a result, more management commands to write the graphs and links are forwarded toward the network devices. The remaining difference could be due to the fact that D-MSR and WirelessHART use different management approaches. Whereas D-MSR relies on the distributed approach, WirelessHART makes use of the centralized management approach, which is far more expensive in terms of time and resources.

#### 6.7.3. Coping with Changes and Disturbances in the Network

**Figure 32.**
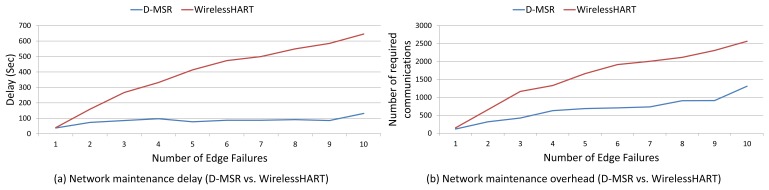
Network maintenance delays (**a**) and overhead (**b**) (D-MSR *vs.* WirelessHART).

In this part, we evaluate the performance of D-MSR in coping with changes in the network. Figure 32 shows the different behavior of D-MSR and WirelessHART in the case of different numbers of edge failures, which are chosen randomly, thereby implying different hop distances from the gateway. We increase the number of edge failures from 1 to 10 and measure the delay, and the number of required communications for coping with edge failure in D-MSR and WirelessHART.

In case of edge failure in D-MSR, the connection manager releases all the reserved communication resources that have failed. The routing layer establishes the new routes then the connection manager re-establishes new connections by reserving resources along the new routes. The delay and overhead in re-establishing connections are shown for both D-MSR and WirelessHART in Figure 32.

In WirelessHART, even though the network may still work through an alternative path when graphs are unreliable, the implemented system management algorithm is set to establish new graph and construct new communication schedule. Moreover, it needs to report the edges failure to the centralized network manager, who subsequently establishes new routes, releases the previous schedules on the old routes and construct new communication schedule (links) on the new routes. In D-MSR, this procedure is done in a distributed manner and this causes the relatively low delay and number of required communications. For example, when the number of broken edges is 10, the number of required communications for network maintenance is on average 48% less for D-MSR, compared to WirelessHART. Furthermore, the network maintenance delay is 79% less.

## 7. Conclusions and Future Work

This paper presented a distributed network management scheme to address the real-time, reliability and throughput requirements of monitoring and process control applications in industrial automation. The resource reservation technique is used in D-MSR for allocating and reserving the communication resources along the path between two end-points (sensors and actuators/gateway). Channel hopping technique is used in D-MSR to prevent external interference and multipath fading in order to provide a reliable communication. This paper showed that D-MSR is more efficient than WirelessHART in managing the network when it comes to node joining, reserving the communication resources (either to exchange management packets or sensor data packets), and coping with network dynamicity (e.g., node or edge failures) in terms of latency and overhead. Furthermore, in case of extensive external interference, D-MSR requires less time to reach the stable data delivery ratio value in comparison with WirelessHART.

The spatial reuse of communication resources in D-MSR improves the throughput in the large-scale network at the potential cost of reduced reliability due to internal interference. That is because concurrent transmissions in the same cell may cause transmission failure even when the edges are two hops away from each other, since in a realistic setting the interference and transmission range may not be equal. On the other hand, by avoiding the spatial reuse of communication resources in WirelessHART, the throughput is reduced. This makes WirelessHART less suitable for large scale networks.

Control in the field is not recommended by WirelessHART network. The network manager in WirelessHART supports peer-to-peer sessions between sensors and actuators if the resulting communications are routed via the gateway. This results in more energy consumption by the nodes close to the access points. D-MSR, On the other hand solves that problem by enabling peer-to-peer sessions communication in the field without involving the gateway or access points. This also results in lower energy consumption over the whole network.

The end-to-end delay in D-MSR is close to that of WirelessHART. This result shows that D-MSR can address the real-time requirements, while also achieving a higher efficiency in the network management than WirelessHART, in terms of delay and overhead. Even though the results are promising already, the following points are expected to improve the capabilities of D-MSR.

### 7.1. Supporting Multipath Mechanism in the D-MSR

To provide reliable communication between the endpoints, multipath routing is considered in the routing layer. In this case if a node fails or an edge is broken, an alternative path can be used for delivering the packets. This approach is followed in several industrial wireless standards such as WirelessHART and ISA100.11a. We intend to consider this capability in the routing layer and in D-MSR.

### 7.2. Avoiding the Spatial Reuse of the Communication Resources and Improving Reliability

In this paper, we assumed that the two hops reuse distance guarantees that concurrent transmissions in the same cell will not cause internal interference. In a realistic setting, however, the interference and transmission range of a node may not be equal. This may cause internal interference between those concurrent transmissions. To improve the reliability of D-MSR, we proposed a solution by considering the *virtual* links that represent the interfering links. We intend to assess another potential solution in which the communication resources (timeslot-channel matrix) are divided into several timeslot-channel blocks and the authority of each block of resources is delegated to different two-hop neighborhoods in a distributed manner. We intend to add this capability to D-MSR to avoid the spatial reuse of communication resources in order to address the requirements of those applications for which improving reliability is more important than losing high throughput.

### 7.3. Applying Reactive Discovery for Point-to-Point Routes

Process closed-loop control applications require peer-to-peer sessions between sensors and actuators. In those applications, sensor data periodically streams from sensors to the actuators without needing to involve the gateway or central controller. The RPL used in the D-MSR routing layer, is not recommended to be used for a peer-to-peer traffic mode. That is because, when sensor and actuator need to communicate, the sensor data are restricted to travel in the “up” direction toward a common ancestor and is then forwarded “down” toward the actuator. This scheme may also result in traffic congestion near the gateway. We intend to use a source-initiated reactive extension of the RPL protocol called P2P-RPL [26] in the D-MSR network layer. P2P-RPL enables the field devices to discover the shorter routes to one or more field devices on demand and addresses the point-to-point traffic model requirements without the mentioned drawbacks.

### 7.4. Supporting Point-to-Multipoint in D-MSR

During the resource reservation in D-MSR, we focus on establishing a point-to-point connection between one sensor and one actuator node. In certain industrial closed-loop control applications involving a sensor and multiple actuators, raw sensor readings are streamed from the sensor to the actuators. In traditional Fieldbus technologies such as Foundation Fieldbus, WorldFIP, and ControlNet, certain sensor nodes (the publishers) produce information that they publish to the network. Other groups of sensors or actuators (the subscribers) that are interested in that information listen to the publishers and update their local copy. This scenario can also occur in the wireless approach. In this case we have to consider establishing a point-to-multipoint connection. A point-to-multipoint connection allows one end point to send its traffic to two or more endpoints. The endpoint which generates the traffic is referred to as the root of the connection, whereas an endpoint that receives this traffic is referred to as a leaf. This feature exists in ATM networks and we intend to use the same concepts to add this capability to D-MSR.

## Figures and Tables

**Table 1.** Different classes of applications as defined by ISA.

**Category**	**Class**	**Application**	**Description**	**Increasing Importance of Message Timeliness ←**

Safety	0	Emergency action	Always critical	

Control	1	Closed-loop regulatory control	Often critical	
	2	Closed-loop supervisory control	Usually noncritical	
	3	Open-loop control	Human in loop	

Monitoring	4	Alerting	Short-term operational consequence	
	5	Logging and downloading/uploading	No immediate operational consequence	

**Table 2.** List of additional information included in the advertisement payload.

**Additional Information**	**Description**
Link table information of the advertiser	Used by receivers to construct the schedule-matrix (Discussed in Section 4.1)
List of advertisement cells used by the advertiser and its neighbors	Used by receivers to select a free advertisement cell (Discussed in Section 4.1)
List of free timeslots of the advertiser	Used by receivers to define initial communication links (Discussed in Section 4.2)

**Table 3.** NS-2 simulation parameters.

**Parameter**	**Value**	**Parameter**	**Value**
Number of nodes	Gateway, two access points, 53 field devices	Radio range	15 meters
Simulation area	100 × 100 m^2^	Frequency Band and channel	2.4 GHz, 11–26 channels
Placement	Regular distribution	Sensor traffic rate	1 per 2 seconds
Data rate	250 kb/s	Application traffic model	CBR

**Table 4.** Interference steps details.

**Interference Steps**	**Affected Edges**
Step-i	19–10, 19–18, 19–20, 19–28, 19–29, 19–30, 21–12, 21–11, 21–20, 21–31, 21–32, 21–22
Step-ii	9–6, 9–8, 9–3, 9–10, 9–18, 9–17, 34–23, 34–35, 34–45, 34–44, 34–43, 34–33
Step-iii	31–30, 31–32, 31–40, 31–41, 24–14, 24–25, 24–36, 24–35, 24–23, 24–13
Step-iv	16–17, 17–27, 27–28, 28–37, 37–38, 26–25, 25–36, 36–35, 35–45, 45–44
Step-v	39–38, 39–29, 39–30, 39–40, 42–41, 42–32, 42–33, 42–43
Step-vi	22–12, 22–13, 22–23, 22–33, 22–32, 20–11, 20–10, 20–31, 20–30

**Table 5.** Periodic messages rates.

**Item**	**Parameter**	**Value**	**Transmission type**
WirelessHART Periodic management data	Health report rate	90 sec	Acknowledged unicast
	Advertisement rate	2 sec	Un-Acknowledged broadcast

D-MSR Periodic management data	RPL control message rate	20 sec	Acknowledged unicast or Broadcast
	Advertisement rate	2 sec	Un-Acknowledged broadcast

Application Data for Both WirelessHART and D-MSR	Sensor Data rate	2 sec	Acknowledged unicast

**Table 6.** Energy-consumption per transaction and its formula.

**Notation**	**Formula**	**Value**
Acknowledged Tx	TsCCA * *Listen power* + *TsMaxPacket* * *Transmit power* + *TsAck* * *Receive power*	303 μJ
Acknowledged Rx	*TsMaxPacket* * *Receive power* + *TsAck* * *Transmit power*	311 μJ
Broadcast Tx	TsCCA * *Listen power* + *TsMaxPacket* * *Transmit power*	252 μJ
Broadcast Rx	*TsMaxPacket* * *Receive power*	264 μJ
Idle Rx	*TsRxWait* * *Listen power*	136 μJ

**Table 7.** Energy-consumption parameters.

**Parameter**	**Value**	**Parameter**	**Value**
Radio chip	CC 2420	TsRxWait	2.2 ms
Transmit power (0 dBm)	57.42 mW	TsAck (26 bytes)	0.832 ms
Receive power	62.04 mW	TsCCA	0.128 ms
Listen power	62.04 mW	TsRxTx (TxRx turnaround)	0.192 ms
TsMaxPacket (133 bytes)	4.256 ms		

**Table 8.** Energy-consumption in the network (in 1,000 s) during normal operation.

	**Item**		**WirelessHART**	**D-MSR**
Static environment	Network management energy		38.77 J	40.70 J
	Application traffic energy		52.84 J	42.71 J
	Total Energy (without idle)		91.61 J	83.41 J
	Idle listening Energy		51.48 J	31.34 J
Dynamic environment (Link failures)	Network maintenance energy	3 Links	0.717 J	0.261 J
		6 Links	1.177 J	0.435 J
		9 Links	1.421 J	0.560 J

**Figure A1.**
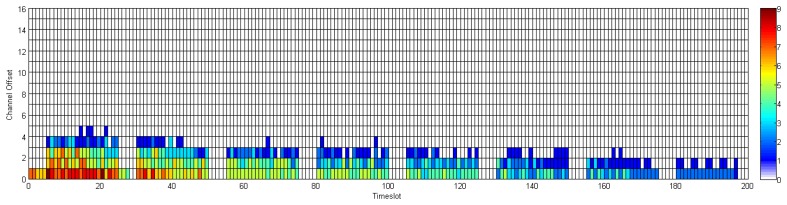
Communication schedule of D-MSR network with seven nodes per one-hop neighborhood.

**Figure A2.**
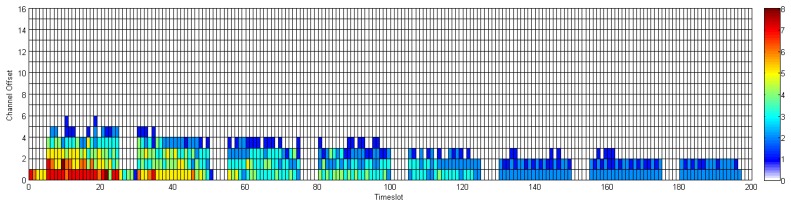
Communication schedule of D-MSR network with nine nodes per one-hop neighborhood.

**Figure A3.**
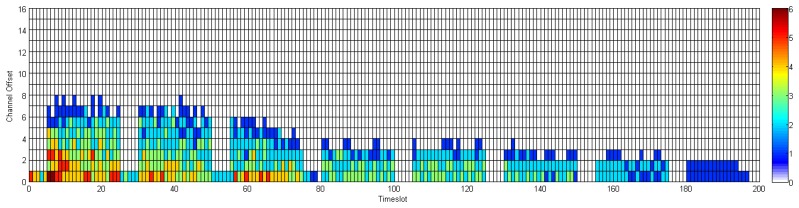
Communication schedule of D-MSR network with 17 nodes per one-hop neighborhood.

**Figure A4.**
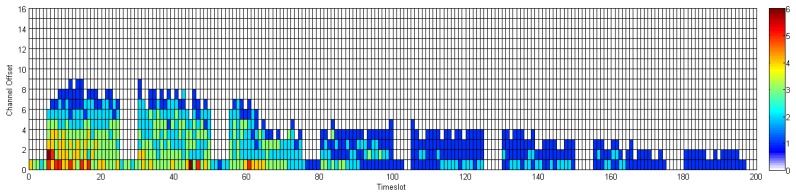
Communication schedule of D-MSR network with 19 nodes per one-hop neighborhood.

**Figure A5.**
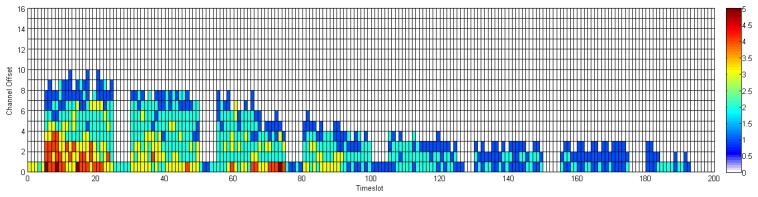
Communication schedule of D-MSR network with 21 nodes per one-hop neighborhood.

**Figure A6.**
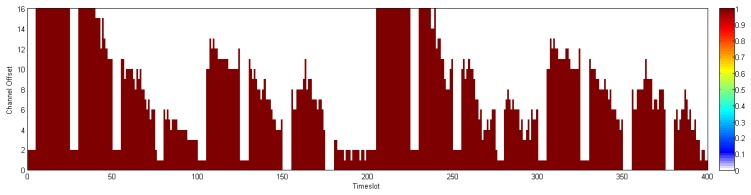
Communication schedule of WirelessHART network with seven nodes per one-hop neighborhood.

**Figure A7.**
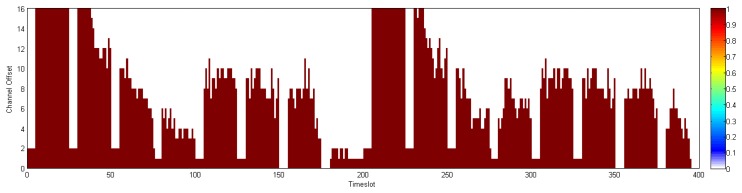
Communication schedule of WirelessHART network with nine nodes per one-hop neighborhood.

**Figure A8.**
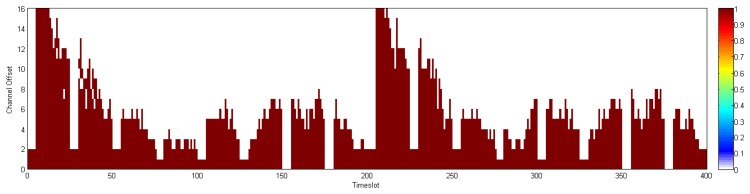
Communication schedule of WirelessHART network with 17 nodes per one-hop neighborhood.

**Figure A9.**
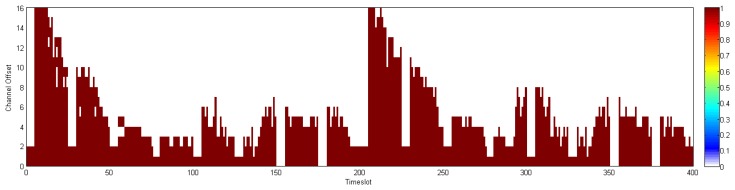
Communication schedule of WirelessHART network with 19 nodes per one-hop neighborhood.

**Figure A10.**
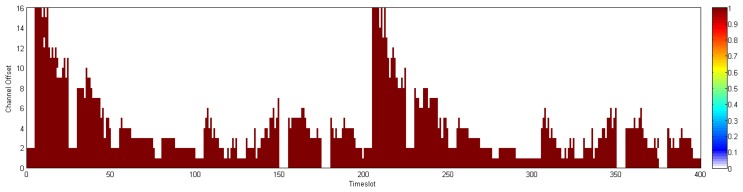
Communication schedule of WirelessHART network with 21 nodes per one-hop neighborhood.

## Appendix

This section provides several figures that show the communication schedule matrices of D-MSR and WirelessHART for different network densities. As discussed in Section 6.4, we gradually increased the network density from 7 to 21 nodes in each one-hop neighborhood in order to assess different communication schedules in different network densities.

## References

[1] Frey J., Lennvall T. (2009). Wireless Sensor Networks for Automation. Embedded Systems Handbook.

[2] Pister K., Thubert P., Dwars S., Phinney T. (2009). Industrial Routing Requirements in Low-Power and Lossy Networks; ROLL RFC 5673. http://www.ietf.org/rfc/rfc5673.txt.

[3] ISA-100.11a-2011 Wireless systems for Industrial Automation: Process Control and Related Applications. http://www.isa.org/Template.cfm?Section=Standards8&template=/Ecommerce/ProductDisplay.cfm&ProductID=11931.

[4] IEC 62591: Industrial Communication Networks - Wireless Communication Network and Communication Profiles - WirelessHART. http://webstore.iec.ch/webstore/webstore.nsf/Artnum_PK/43964.

[5] ZigBee Specification. http://www.zigbee.org/Specifications.aspx.

[6] Christin D., Mogre P.S., Hollick M. (2010). Survey on wireless sensor network technologies for industrial automation: The security and quality of service perspectives. Future Internet.

[7] Zand P., Chatterjea S., Das K., Havinga P. (2012). Wireless industrial monitoring and control networks: The journey so far and the road ahead. J. Sens. Actuat. Netw..

[8] Watteyne T., Lanzisera S., Mehta A., Pister K.S.J. Mitigating Multipath Fading through Channel Hopping in Wireless Sensor Networks.

[9] (2006). IEEE Standard for Information Technology—Local and metropolitan area networks—Specific requirements– Part 15.4: Wireless Medium Access Control (MAC) and Physical Layer (PHY) Specifications for Low Rate Wireless Personal Area Networks (WPANs).

[10] (2012). IEEE Standard for Local and metropolitan area networks—Part 15.4: Low-Rate Wireless Personal Area Networks (LR-WPANs) Amendment 1: MAC sublayer.

[11] Zand P., Chatterjea S., Ketema J., Havinga P. A Distributed Scheduling Algorithm for Real-Time (D-SAR) Industrial Wireless Sensor and Actuator Networks.

[12] (1995). ATM Forum Technical Committee, ATM user-network interface (UNI) specification version 3.1.

[13] Winter T., Thubert P., Team R.A. (2012). RPL: IPv6 routing protocol for low power and lossy networks, RFC 6550. IETF ROLL WG Tech. Rep..

[14] Pister K., Doherty L. TSMP: Time synchronized mesh protocol.

[15] Song H., Xiuming Z., Mok A.K., Deji C., Nixon M. Reliable and Real-Time Communication in Industrial Wireless Mesh Networks.

[16] Badia L., Erta A., Lenzini L., Zorzi M. (2008). A general interference-aware framework for joint routing and link scheduling in wireless mesh networks. Network IEEE.

[17] Gupta P., Kumar P.R. (2000). The capacity of wireless networks. Inf. Theory IEEE Trans..

[18] Munir S., Lin S., Hoque E., Nirjon S.M.S., Stankovic J.A., Whitehouse K. Addressing Burstiness for Reliable Communication and Latency Bound Generation in Wireless Sensor Networks.

[19] Suriyachai P., Brown J., Roedig U., Rajaraman R., Moscibroda T., Dunkels A., Scaglione A. (2010). Time-Critical Data Delivery in Wireless Sensor Networks. Distributed Computing in Sensor Systems.

[20] Salajegheh M., Soroush H., Kalis A. HYMAC: Hybrid TDMA/FDMA Medium Access Control Protocol for Wireless Sensor Networks.

[21] Ergen S.C., Varaiya P. (2006). PEDAMACS: Power efficient and delay aware medium access protocol for sensor networks. IEEE Trans. Mobile Comput..

[22] Van Hoesel L.F., Havinga P. A Lightweight Medium Access Protocol (LMAC) for Wireless Sensor Networks: Reducing Preamble Transmissions and Transceiver State Switches.

[23] Tinka A., Watteyne T., Pister K., Zheng J., Simplot-Ryl D., Leung V.C.M. (2010). A Decentralized Scheduling Algorithm for Time Synchronized Channel Hopping Ad Hoc Networks. Ad Hoc Networks.

[24] Zand P., Chatterjea S., Ketema J., Havinga P. (2011). D-SAR: A Distributed Scheduling Algorithm for Real-time, Closed-Loop Control in Industrial Wireless Sensor and Actuator Networks. http://eprints.eemcs.utwente.nl/20078.

[25] Zand P., Dilo A., Havinga P. Implementation of WirelessHART in NS-2 Simulator.

[26] Philipp M., Martocci J., Brandt A., Baccelli E., Goyal M. Reactive Discovery of Point-to-Point Routes in Low Power and Lossy Networks. IETF Internet Draft draft-ietf-roll-p2p-rpl-17. http://tools.ietf.org/html/draft-ietf-roll-p2p-rpl-17.

